# Stability of gabapentin in extemporaneously compounded oral suspensions

**DOI:** 10.1371/journal.pone.0175208

**Published:** 2017-04-17

**Authors:** Mihaela Friciu, V. Gaëlle Roullin, Grégoire Leclair

**Affiliations:** Faculté de pharmacie, Université de Montréal, Montréal, QC, Canada; Institute of medical research and medicinal plant studies, CAMEROON

## Abstract

This study reports the stability of extemporaneously prepared gabapentin oral suspensions prepared at 100 mg/mL from bulk drug and capsules in either Oral Mix or Oral Mix SF suspending vehicles. Suspensions were packaged in amber plastic bottles and amber plastic syringes at 25°C / 60%RH for up to 90 days. Throughout the study period, the following tests were performed to evaluate the stability of the preparations: organoleptic inspection to detect homogeneity, color or odor changes; pH measurements; and gabapentin assay using a stability-indicating HPLC-UV method. As crystallization was observed at 5°C, storage at this temperature condition is not recommended. All preparations stored at 25°C / 60%RH remained stable for the whole study duration of 90 days.

## Introduction

Gabapentin is an antiepileptic drug. Its mechanism of action is not fully understood, but it could increase the synthesis of GABA in the brain by promoting the activity of glutamate decarboxylase and reducing the activity of branched-chain amino acid aminotransferase [1]. The net effect would be a reduced synthesis of glutamate. Gabapentin also binds to voltage-operated N and P/Q calcium channels causing the inhibition of voltage-dependent calcium current [1]. This mechanism of action is different from other classes of antiepileptic drugs that increase GABA-mediated events, block sodium channels or inhibit glutamate mediated event.

Gabapentin is commercialized under the name Neurontin by Pfizer in most regions of the world. Numerous generic versions of this drug are also available. In the USA, this drug is available as capsules and tablets containing 100, 300, 400, 600 or 800 mg of gabapentin (C_9_H_17_NO_2_, 171.24 g/mol) as well as an oral solution containing 50 mg/mL. According to product label of Neurontin in the USA, this drug is indicated for the treatment of post-herpetic neuralgia in adults as well as an adjunctive treatment of partial onset seizures in adults and patients 3 years and older [2]. This drug need to be titrated over a period of three days up to a maintenance dose of 600 mg three time a day for patient of 12 years and older [2]. The maintenance dose in children of 3 to 12 years of age is 10 to 15 mg/kg/day divided in three equal doses per day [2].

The Canadian Pain Society considers gabapentin to be a first line analgesic in the management of chronic neuropathic pain [3]. Recently, gabapentin was used in the management of numerous conditions in children including insomnia associated with neurodegenerative disorders [4], post-traumatic headaches [5] and visceral hyperalgesia associated with neurologic or gastrointestinal morbidities [6].

Unfortunately, gabapentin has not received an approved indication in patient of less than 18 years of age in Canada and it is only available as solid oral dosage forms in this country [7]. The extemporaneous preparation of a compounded oral solution by the pharmacist is therefore required in order to administer this drug to children.

The biopharmaceutical properties of gabapentin have been reported [8]. Its absorption is not limited by its solubility which is above 100 mg/mL [8]. Also, as a large neutral amino acid, gabapentin is a substrate of the system-L transporter family [8]. This mechanism of absorption could be saturable as the steady-state plasmatic concentration is no longer linear for doses larger than 1800 mg/day [8]. Absolute bioavailability is about 80% at 300 mg/day and decreases to 27% when doses of 4800 mg/day were administered [8].

Solid oral dosage forms of gabapentin are immediate release capsules and immediate-release tablets [2]. The liquid form available in the USA is a simple aqueous solution containing glycerin, xylitol, purified water and artificial flavor [2]. This solution requires refrigeration [2]. Considering its biopharmaceutical properties, a compounded liquid formulation of gabapentin should be equivalent to the commercial products as long as it is compounded as a solution. The absence of precipitation and no change in potency would be two critical quality attributes that will need to be controlled during the shelf life of this compounded product. Storage temperature also need to be evaluated; as stability of compounded preparations is usually evaluated under a shorter period of time (3 months) compared to commercial preparations (2 to 5 years), storage for 3 months at controlled room temperature should also be considered in the stability protocol.

With regards to compounded preparations, stability is generally defined as the absence of organoleptic changes, the absence of physical changes, a difference of pH of not more than 0.5 compared to the initial pH as well as a drug substance concentration of not less than 90.0% relative to the initial concentration [9–16].

A few studies reported the stability of compounded oral suspensions of gabapentin. In 1999, Nahata evaluated preparations of gabapentin 100 mg/mL compounded from gabapentin capsules in both methylcellulose 1% and a mixture of Ora Plus and Ora Sweet (1:1) conditioned in plastic bottles [17]. These preparations remained stable at least 91 days at 4°C and at least 56 days at 25°C [17]. In 2012, Sorenson reported that gabapentin 50 mg/mL in SyrSpend SF conditioned in low actinic bottles was stable at least 90 days when refrigerated or stored at room temperature [18]. In 2014, Soliman tested the stability of gabapentin 100 mg/mL suspension in a methylcellulose vehicle after three months of refrigeration. The assay of this preparation fell from 95.1 mg/mL initially to 69.9 mg/mL after three months (73.5% of initial concentration) [19]. This result was unexpected considering that Nahata and Sorenson previously demonstrated stability under similar conditions.

Methylcellulose vehicle is cumbersome to prepare, while SyrSpend SF is not readily available in Canada. Furthermore, conflicting information is available regarding the stability of compounded preparations of gabapentin under refrigerated conditions.

The objective of this study was therefore to unequivocally evaluate the stability of compounded liquid preparations of gabapentin in refrigerated conditions, as well as to study the stability of this compound in a pediatric vehicle readily available in Canada. We applied the generally-agreed definition of stability as previously described. In order to minimize the volume of administration, a concentration of 100 mg/mL was selected as the aqueous solubility was reported higher than this value [8]. Furthermore, a stability-indicating HPLC-UV method for the quantification of gabapentin needed to be developed and validated in order to achieve the primary objective of this study.

In this study, we first evaluated the effect of refrigeration on multiple gabapentin preparations. We then report a comprehensive evaluation of the stability of gabapentin compounded preparations (100 mg/mL) using a validated stability-indicating HPLC-UV method. We studied the effect of the following variables: (1) time: up to 90 days; (2) temperature: 25°C; (3) gabapentin source: bulk drug and capsules; (4) vehicle: Oral Mix and Oral Mix SF; and (5) packaging: amber PET bottles and oral plastic syringes.

## Materials and methods

### Materials

A single lot of gabapentin 300 mg capsules (lot KV4024, exp 2016–02, Apotex Canada) and a single lot of gabapentin USP bulk drug powder (lot 49895/G, exp 2016–02, Medisca Pharmaceutique Inc., Montréal, QC, Canada) were used to develop and validate the analytical method as well as conduct the stability study. The experiments conducted to evaluate the impact of refrigeration where performed using gabapentin 300 mg capsules (lot MR5496, exp 2018–03, Apotex Canada) and gabapentin USP bulk drug powder (lot 609265/E, exp 2018–06, Medisca Pharmaceutique Inc., Montréal, QC, Canada). Gabapentin USP bulk drug powder, Oral Mix, Oral Mix SF, 50 mL amber PET bottles with black phenolic caps as well as PreciDose Dispenser 1-mL amber oral syringes were graciously provided by Medisca Pharmaceutique Inc. Acetonitrile and methanol of HPLC grade as well as sodium hydroxide solution (1 M) were purchased from Fisher Scientific Canada. Monobasic potassium phosphate was purchased from J.T. Baker Chemicals (distributed by ACS Chemicals, Montréal, QC, Canada). Water was purified using a Milli-Q Synthesis A10 system (Millipore, Etobicoke, ON, Canada). An aqueous phosphate buffer was prepared by dissolving monobasic potassium phosphate in water (10 mM); the pH was adjusted to 6.2 using an aqueous NaOH solution (1 M). The HPLC mobile phase consisted of a mixture of acetonitrile and phosphate buffer (84:16 v:v).

### Compounded preparations from bulk drug powder

The same procedure was used to prepare gabapentin suspensions (100 mg/mL) in both Oral Mix and Oral Mix SF from bulk drug powder. Gabapentin USP (20 g) was accurately weighed and transferred into a mortar. The drug powder was then mixed using either Oral Mix or Oral Mix SF (5 mL) until forming a smooth paste. Additional amounts of vehicle were then incorporated in increments to complete the preparation to the required volume (200 mL).

### Compounded preparations from capsules

Similarly compounded suspensions (100 mg/mL) were prepared from gabapentin 300 mg capsules in Oral Mix and Oral Mix SF. The content of the capsules (70 × 300 mg capsules) was a coarse powder that was reduced into a fine powder using a pestle in a mortar. This fine powder was then wetted using the selected vehicle (15 mL). Additional amounts of vehicle were then incorporated in increments to complete the preparation to the required volume (210 mL).

### Effect of refrigeration on gabapentin liquid preparations

Gabapentin (100 mg/mL) liquid preparations made from bulk drug powder as well as capsules in Oral Mix and Oral Mix SF was produced as described above. Gabapentin (100 mg/mL) liquid preparations made from capsules in OraBlend (1:1 mixture of OraPlus and OraSweet) and in methylcellulose 1% were produced as described by Nahata [17]. A gabapentin (50 mg/mL) liquid preparation made from bulk drug in SyrSpend SF was produced as described by Sorenson [18]. Gabapentin preparations as well as their corresponding vehicles were poured in 20-mL clear scintillation vials and stored at 5°C ± 2°C (n = 3 for each preparation). Each vial was evaluated after one and seven days by visual examination. Eventual precipitates were examined by optical microscopy (Zeiss Discovery V8 SteREO microscope, total magnification 6x).

### Stability study design

Each different preparation was packaged in 50-mL amber plastic bottles (filling volume of 30 mL, three bottles) and 1-mL amber plastic syringes (filling volume of 1 mL, 24 syringes). Containers were then stored at 25°C ± 2°C / 60% ± 5%RH (Thermo Scientific, Forma Environmental Chamber, OH, USA). The stability study was designed in order to replicate reactions of interest in at least three containers for each specific condition.

At predetermined time points (0, 7, 14, 30, 45, 60, 75 and 90 days), 1 mL was retrieved from each bottle and three syringes were pulled out. Prior to sampling, bottles were shaken and the suspensions inspected for consistency, color and odor changes; sample was then transferred to a 1.5-mL centrifuge tube for further analysis. In the case of syringes, the organoleptic properties were verified after the transfer of the suspension into 1.5-mL centrifuge tubes. The pH of each sample was evaluated using a pH meter (pH 211, Hanna Instruments, Montréal, QC, Canada) and the gabapentin concentration was evaluated using a validated stability-indicating HPLC-UV method. This study design ensured that ageing conditions were replicated in at least three separate containers.

### Sample preparation for HPLC injection

Methanol (450 μL) was added to the test gabapentin suspension (100 mg/mL, 50 μL) in a 1.5-mL centrifuge tube. The tube was vigorously vortexed (20 s) and then centrifuged (9,400 g, 15 min). This resulted in a phase separation; part of the vehicle excipient precipitated. An aliquot of the supernatant (100 μL) was then transferred into a 1.5-mL centrifuge tube and diluted using HPLC mobile phase (300 μL). The tube was vigorously mixed using a vortex (10 s), transferred to a 96-well plate and then analyzed using a validated stability-indicating HPLC-UV method.

### Preparation of forced degradation samples

Hydrolytic degradation of gabapentin was evaluated by diluting a gabapentin suspension in Oral Mix (100 mg/mL, 0.5 mL) with water (0.5 mL), aqueous NaOH (0.1 M, 0.5 mL), aqueous HCl (0.1 M, 0.5 mL) or aqueous hydrogen peroxide (3%, 0.5 mL). These solutions were heated 4 h at 80°C and brought back to room temperature. Sample preparation for HPLC injection was then performed as described above prior to analysis using the HPLC-UV method.

### HPLC-UV method

The HPLC instrumentation (Prominence UFLC, Shimadzu, Laval, QC, Canada) consisted of an LC-20AD binary pump, a DGU-20A5 solvent degasser, a SPD-M20A multiple wavelength photodiode array detector set at 210 nm, a SIL-20AC HT refrigerated autosampler set at 25°C, a CTO-20AC column oven set at 40°C and a YMC-Pack NH2 column (100 × 3.0 mm, 5 μm, 12 nm, YMC America, Allentown, PA, USA). Mobile phase was eluted isocratically at a flow rate of 0.8 mL/min. Gabapentin peak had a retention time of about 2.9 min; area of this peak was used to perform the quantification. Injections were duplicated for test samples and triplicated for standard samples.

Calibration was performed with gabapentin bulk powder in Oral Mix and Oral Mix SF. A standard suspension was prepared using gabapentin (500 mg) in both vehicles (5 mL). Factoring in the displacement produced by the gabapentin powder (0.796 mL/g in Oral Mix and 0.762 mL/g in Oral Mix SF), the nominal concentrations of these standard suspensions were 0.926 mg/mL for Oral Mix and 0.929 mg/mL for Oral Mix SF. These standard suspensions (500 μL) were diluted using methanol (4,500 μL), vigorously vortexed (30 s) and then centrifuged (9,400 g, 15 min). Resulting supernatants were serially diluted using mobile phase to achieve 5.00, 3.75, 2.50, 1.00 and 0.50 mg/mL. This calibration procedure covered a range of about 20% to 185% of the target concentration; mobile phase was used as blank sample (0% of target concentration). All calibration samples were injected in triplicate at the time of sample quantification. For each run of analyses, system suitability samples (quality control standards, 2.5 mg/mL) were injected every 24 injections. System suitability was established on resolution, tailing factor and number of theoretical plate, as calculated with the help of the HPLC software (LabSolutions 5.54 sp5, Shimadzu Corporation).

## Results

### Effect of refrigeration on gabapentin liquid preparations

**Fig 1:**
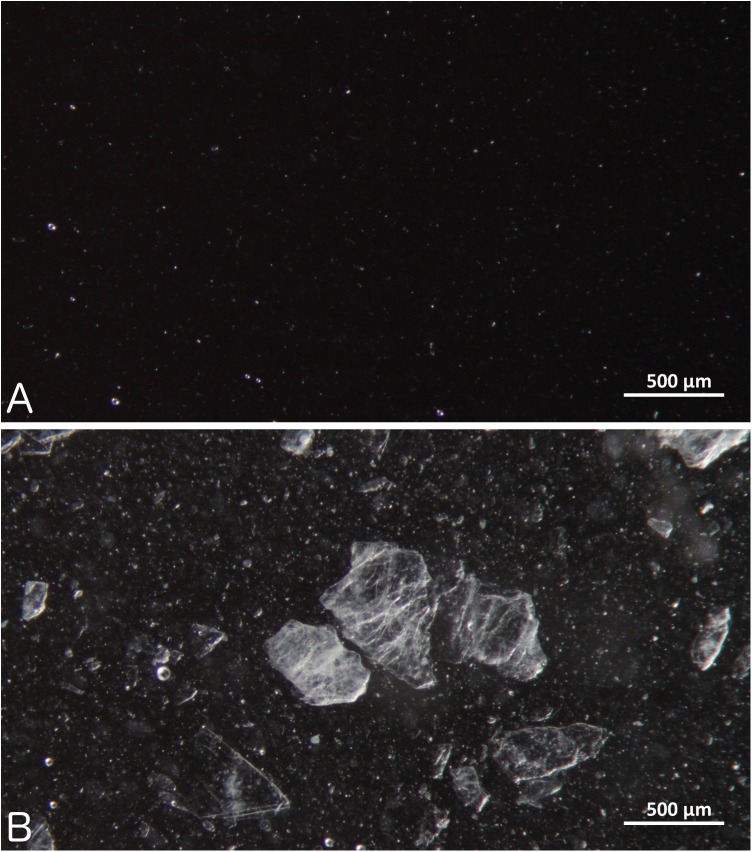
Microscopic appearance of gabapentin suspension 100 mg/mL prepared from capsules using Oral Mix. (A) Immediately after preparation; and (B) after 14 days of storage at 5°C.

All tested blank vehicles were opalescent, but methylcellulose that was a clear solution. When gabapentin liquid preparations were produced in these vehicles, there was little or no change in appearance suggesting that most of the drug was in the dissolved state. In the case of methylcellulose, it could be observed that some drug remained in the solid state as the preparation became opalescent. It should be noted that all these preparations contained 100 mg/mL, but SyrSpend that contained 50 mg/mL. After 1 day, precipitation could be observed directly with the unaided eye for the preparations made from capsules in OraBlend and in methylcellulose (S1 Table). After 7 days, precipitation was also observed in the gabapentin preparations made from bulk drug and capsules in Oral Mix and from capsules in Oral Mix SF. No precipitation could be observed in the preparations made from bulk drug in Oral Mix SF as well as bulk drug in SyrSpend. Fig 1 illustrates a typical crystal obtained when precipitation was observed. In some cases particles as large as a few millimetres were observed.

### Validation of the HPLC-UV method

This method was validated based on the United States pharmacopeia guidelines. The recommendations for analytical procedures for the quantitation of major components of active ingredients in finished pharmaceutical products (category I) was followed [20]. According to these recommendations, the validation procedure should include the following points: Accuracy, precision, specificity, linearity and range. Validation was performed using the standard calibration samples in both vehicles over the range of 20% to 185% of target concentration.

Accuracy as defined by the relative error between the regressed concentration and the true concentration was calculated for each point the calibration curve. This error was not more than 1.3% for all tested conditions.

Precision of the analytical method including sample preparation was also evaluated. At time zero of the stability study, four gabapentin suspensions (100 mg/mL) were prepared from capsule or bulk powder in Oral Mix or Oral Mix SF. These four suspensions were sampled six times and sample preparation was performed on each of these. The coefficient of variation of the responses obtained for each of these samples for HPLC injection was comprised between 0.8% and 1.9%.

Intermediate precision as defined by the precision over several days was also evaluated. A standard solution of gabapentin was prepared at target concentration and was injected every thirty injections of HPLC injection sequences. Signals obtained from these injections throughout the 90-day study were used to calculate an intermediate precision of 2.7% (n = 11) and 0.5% (n = 12) in Oral Mix and Oral Mix SF, respectively.

Precision of the HPLC instrumentation was evaluated using the coefficient of variation measured between replicated injections. This was evaluated using the calibrations performed at time zero of the stability study. For both Oral Mix and Oral Mix SF, this coefficient of variation was not more than 0.8% at all tested concentrations and 0.4% at target concentration.

**Fig 2:**
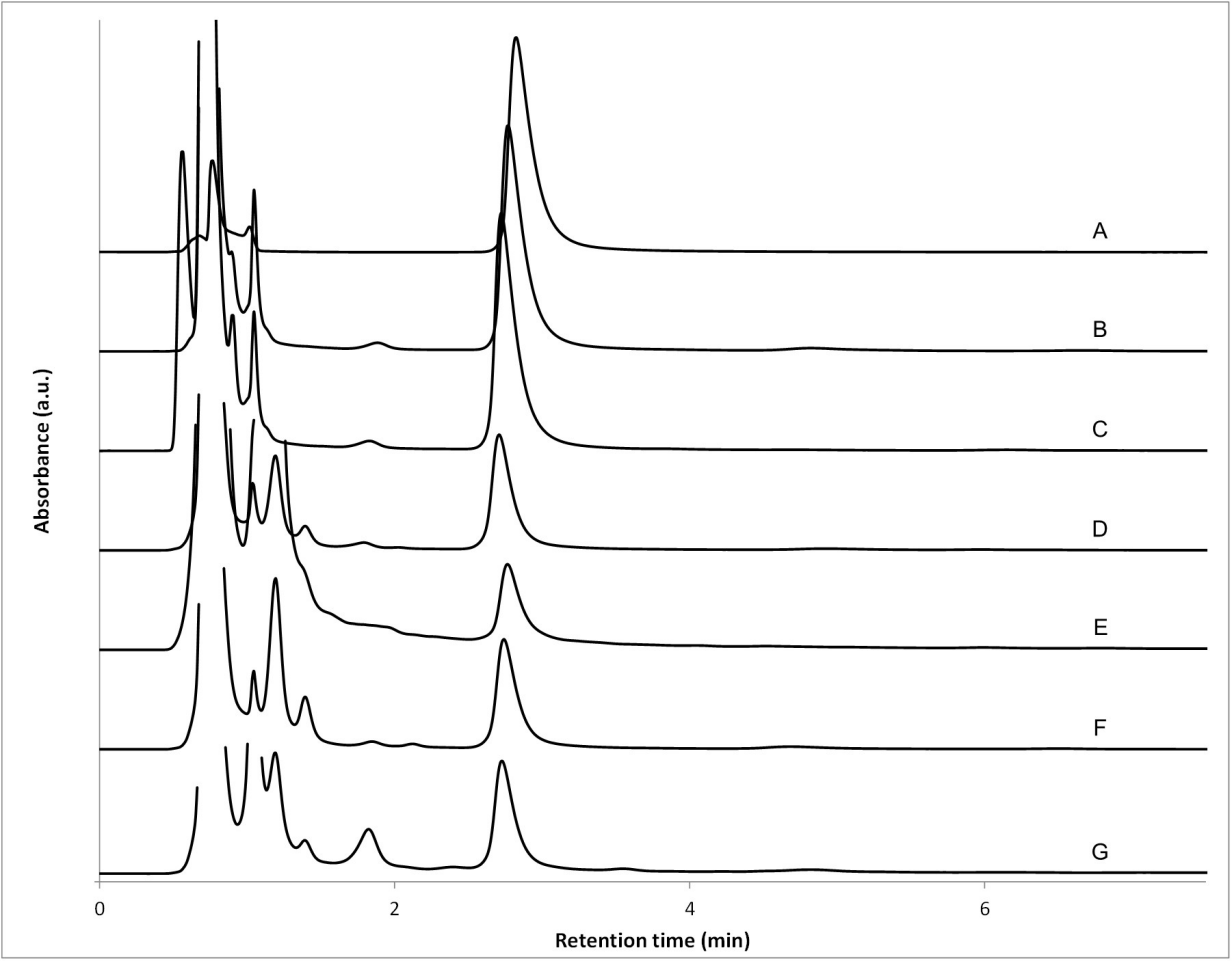
Representative chromatograms. (A) Gabapentin standard solution in mobile phase (2.5 mg/mL); (B) gabapentin suspension prepared form bulk drug using Oral Mix vehicle and submitted to sample preparation for HPLC injection (100 mg/mL suspension, 2.5 mg/mL after sample preparation); (C) gabapentin suspension prepared form bulk drug using Oral Mix SF and submitted to sample preparation for HPLC injection (100 mg/mL suspension, 2.5 mg/mL after sample preparation); gabapentin stressed sample after 4 h at 80°C in (D) water; (E) 0.1 N NaOH; (F) 0.1 N HCl; and (G) 3.0% H_2_O_2_. All stress samples were prepared to have a target concentration of 2.5 mg/mL in the absence of degradation.

Specificity of the method is illustrated in Fig 2. No peak overlap was observed between gabapentin and the ingredients contained in Oral Mix (B) and Oral Mix SF (C). Degradations of 34% and 14% were observed in alkaline (E) and oxidative (G) forced degradation conditions, respectively. Minor degradation of gabapentin was observed in neutral (D, 3%) and acidic (F, 7%) forced degradation conditions. In all cases, no peak overlap was observed between the gabapentin peak, its degradation products and the excipients. This is further supported by the calculation of the gabapentin peak purity index which was not less than 0.9999 when calculated between 190 and 240 nm for each of these chromatograms.

The suitability of the HPLC system was ascertained by numbers of theoretical plates not less than 15,000 (Oral Mix: 22,127 ± 2,629; Oral Mix SF: 15,375 ± 1,477), tailing factors not greater than 2 (Oral Mix: 1.37 ± 0.04; Oral Mix SF: 1.69 ± 0.09) and a resolution, calculated from the degradation study, not less than 1.5 (degradation in acidic conditions, 6.2; in basic conditions: 1.5; in oxidative conditions: 3.4).

Linearity of the method was evaluated by linear regression of the responses obtained after the injection of the calibration samples. For both Oral Mix and Oral Mix SF, r^2^ was not less than 0.9999 over the domain of tested concentrations.

### Stability study

At 25°C for all time points, no crystal growth was observed and organoleptic properties remained constant. The pH of all preparations varied between 5.4 and 5.5. As listed in Tables 1–4, the initial content assay of the preparations was comprised between 101.0 and 106.8 mg/mL. During the 90 days of the stability study at 25°C, gabapentin content assay remained not less than 90.0% of the initial content assay.

**Table 1:** Stability of gabapentin 100 mg/mL formulation prepared from bulk powder using Oral Mix vehicle and stored at 25°C.

	Amber plastic bottles		Amber plastic syringes	
Days	Assay ± SD (mg/mL)	% of initial ± SD	Assay ± SD (mg/mL)	% of initial ± SD
0	101.0 ± 0.9	N/A	101.0 ± 0.9	N/A
7	100.1 ± 1.0	99.2 ± 1.0	100.9 ± 1.1	100.0 ± 1.1
14	95.6 ± 2.0	94.7 ± 2.0	95.4 ± 0.8	94.5 ± 0.8
30	99.2 ± 0.6	98.3 ± 0.6	99.3 ± 0.5	98.3 ± 0.5
45	97.8 ± 3.2	96.9 ± 3.2	97.4 ± 2.1	96.4 ± 2.1
60	95.6 ± 1.1	94.7 ± 1.0	98.6 ± 1.4	97.7 ± 1.4
75	96.5 ± 0.3	95.6 ± 0.3	96.5 ± 0.4	95.6 ± 0.4
90	97.1 ± 0.7	96.2 ± 0.7	96.3 ± 2.1	95.3 ± 2.0

**Table 2:** Stability of gabapentin 100 mg/mL formulation prepared from capsules using Oral Mix vehicle and stored at 25°C.

	Amber plastic bottles		Amber plastic syringes	
Days	Assay ± SD (mg/mL)	% of initial ± SD	Assay ± SD (mg/mL)	% of initial ± SD
0	101.3 ± 1.9	N/A	101.3 ± 1.9	N/A
7	96.7 ± 1.4	95.5 ± 1.3	96.7 ± 2.6	95.5 ± 2.6
14	92.9 ± 1.3	91.7 ± 1.3	93.3 ± 2.2	92.1 ± 2.1
30	100.2 ± 0.3	99.0 ± 0.3	99.7 ± 0.8	98.5 ± 0.8
45	98.6 ± 0.8	97.4 ± 0.8	96.7 ± 2.1	95.5 ± 2.0
60	96.7 ± 2.0	95.5 ± 2.0	95.3 ± 1.1	94.1 ± 1.1
75	96.4 ± 0.9	95.2 ± 0.9	97.1 ± 1.1	95.8 ± 1.1
90	94.3 ± 0.4	93.1 ± 0.4	96.4 ± 1.8	95.2 ± 1.8

**Table 3:** Stability of gabapentin 100 mg/mL formulation prepared from bulk powder using Oral Mix SF vehicle and stored at 25°C.

	Amber plastic bottles		Amber plastic syringes	
Days	Assay ± SD (mg/mL)	% of initial ± SD	Assay ± SD (mg/mL)	% of initial ± SD
0	106.8 ± 0.9	N/A	106.8 ± 0.9	N/A
7	107.7 ± 0.8	100.8 ± 0.8	108.3 ± 1.5	101.4 ± 1.4
14	110.9 ± 2.6	103.8 ± 2.4	109.6 ± 4.2	102.5 ± 3.9
30	111.5 ± 1.4	104.3 ± 1.3	110.6 ± 1.1	103.5 ± 1.0
45	104.7 ± 1.3	98.0 ± 1.2	106.9 ± 1.2	100.0 ± 1.2
60	108.4 ± 2.7	101.4 ± 2.6	105.4 ± 0.4	98.6 ± 0.4
75	113.0 ± 4.0	105.7 ± 3.8	109.9 ± 0.6	102.9 ± 0.5
90	110.7 ± 0.5	103.6 ± 0.5	110.2 ± 1.1	103.1 ± 1.1

**Table 4:** Stability of gabapentin 100 mg/mL formulation prepared from capsules using Oral Mix SF vehicle and stored at 25°C.

	Amber plastic bottles		Amber plastic syringes	
Days	Assay ± SD (mg/mL)	% of initial ± SD	Assay ± SD (mg/mL)	% of initial ± SD
0	105.7 ± 0.8	N/A	105.7 ± 0.8	N/A
7	105.9 ± 2.1	100.2 ± 2.0	107.8 ± 0.7	102.0 ± 0.6
14	107.4 ± 2.8	101.6 ± 2.7	112.8 ± 1.6	106.8 ± 1.5
30	112.2 ± 4.4	106.2 ± 4.1	110.1 ± 1.9	104.2 ± 1.8
45	107.1 ± 0.9	101.4 ± 0.8	105.7 ± 0.6	100.0 ± 0.5
60	105.2 ± 0.3	99.5 ± 0.3	106.0 ± 0.3	100.3 ± 0.3
75	112.0 ± 0.3	105.9 ± 0.3	110.0 ± 1.0	104.1 ± 1.0
90	110.7 ± 0.2	104.7 ± 0.2	110.0 ± 1.6	104.1 ± 1.5

## Discussion

The experiment conducted at 5°C demonstrated that precipitation is a high risk for liquid preparations of gabapentin of 100 mg/mL. Most likely, the solubility of gabapentin decreases at lower temperatures and falls below 100 mg/mL in refrigerated condition. Therefore, preparations of gabapentin 100 mg/mL compounded in Oral Mix and Oral Mix SF should not be refrigerated. However, refrigeration is the recommended storage conditions for Neurontin 50 mg/mL, the commercial gabapentin solution available in the USA [2]. Also, Nahata and Sorenson demonstrated the stability of gabapentin preparations under refrigerated conditions for three months. These preparations of contained 100 mg/mL [17] and 50 mg/mL [18] of gabapentin, respectively. Nonetheless, Soliman showed that a 100 mg/mL preparation was not stable over a period of three months [19]. All these gabapentin liquid formulations were prepared in aqueous vehicles, but exact compositions and gabapentin concentrations varied. Experimental aqueous solubility of gabapentin is reported to be 4.49 mg/mL from DrugBank [21] and over 100 mg/mL from another source [8]. We were able to completely dissolve gabapentin in pure water at a concentration of 100 mg/mL.

Nahata and Soliman reported different results regarding a 100 mg/mL suspension in methylcellulose. It was reported stable for at least 91 days by Nahata (1999), while Soliman measured only 73.5% of the initial content assay under similar conditions. We believe sampling procedure may explain at least in part these different results as a large sample will less likely be affected by the inhomogeneity of the suspension. We also observed that crystal growth rate was different between preparations, some preparations showing millimeter size crystals and other showing smaller crystals.

Regarding the stability study conducted at 25°C, all stability requirements were met for all the preparations made from Oral Mix and Oral Mix SF during the whole study. Of all the studied variables, only temperature had an observable impact. It should be noted that this study only evaluated the variability introduced by the selection of the gabapentin source: commercially-available USP capsules vs. USP bulk drug powder. Although the stability study was performed in at least three separate containers for each condition, manufacturing was performed only once per formulation. No impact of using capsules vs. bulk drug could be observed. As acceptable preparations were obtained in both cases, it appears that this formulation is robust to the gabapentin origin. Our study is thus clinically useful as it reports a stable gabapentin liquid preparation in easy-to-work-with and globally available vehicles that can be stored at room temperature.

## Conclusions

Compounded preparations of gabapentin 100 mg/mL prepared using Oral Mix or Oral Mix SF, bulk drug powder or capsules and packaged in amber PET bottles or amber oral syringes remained stable for at least 90 days at 25°C. As physical changes were observed at 5°C, these preparations should be protected from cold and should not be frozen. Preparations should always be shaken prior use.

## Supporting information

S1 Table Suspension stability in various vehicles, in refrigerated conditions. (DOCX)

S1 Appendix HPLC Results. Excel file containing all reported HPLC results.
(XLSX)

S2 Appendix Browsable Stability Results. Archive containing the HPLC stability results as browsable html pages.
(ZIP)
