# Supplementary material for: Stability of gabapentin in extemporaneously compounded oral suspensions
Source: PLoS One. 2017 Apr 17;12(4):e0175208. doi: 10.1371/journal.pone.0175208 (PMC5393583; doi:10.1371/journal.pone.0175208)
Supplement: S1 Table — (DOCX) [file pone.0175208.s001.docx]

| Gabapentin conc.  (mg/mL) | Drug source | Suspension vehicle | Visual appearance | |
| --- | --- | --- | --- | --- |
|  |  |  | Initial | After  7 days at 5°C |
| 100 | b | Oral Mix | ✓ | Precipitation + |
|  | b | Oral Mix SF | ✓ | ✓ |
|  | c | Oral Mix | ✓ | Precipitation + |
|  | c | Oral Mix SF | ✓ | Precipitation ++ |
|  | c | OraBlend | ✓ | Precipitation +++ after 24 h |
|  | c | Simple syrup / 1% methylcellulose | Opalescent | Precipitation +++ after 24 h |
| 50 | b | SyrSpend SF | ✓ | ✓ |

b: bulk drug powder; c: capsules; ✓: same as blank vehicle; precipitation +: presence of particles could be observed after careful examination; precipitation ++: presence of particles could be easily observed; precipitation +++: presence of particles was obvious.
