# Supplementary material for: Stability of gabapentin in extemporaneously compounded oral suspensions
Source: PLoS One. 2017 Apr 17;12(4):e0175208. doi: 10.1371/journal.pone.0175208 (PMC5393583; doi:10.1371/journal.pone.0175208)
Supplement: S2 Appendix — Archive containing the HPLC stability results as browsable html pages. (ZIP) [file pone.0175208.s003.zip › gaba_s2_html_results/gabapentin.html]

Stability Study Cruncher


### Preparation: bulk-oralmix, Lot: a, Condition: bottle-25

| Days | Assay (mg/mL) | | | % of initial | | | n |  |
| --- | --- | --- | --- | --- | --- | --- | --- | --- |
| 0 | 101.0 | ± | 0.9 |  |  |  | 12 | time zero |
| 7 | 100.1 | ± | 1.0 | 99.2 | ± | 1.0 | 6 | time point |
| 14 | 95.6 | ± | 2.0 | 94.7 | ± | 2.0 | 6 | time point |
| 30 | 99.2 | ± | 0.6 | 98.3 | ± | 0.6 | 6 | time point |
| 45 | 97.8 | ± | 3.2 | 96.9 | ± | 3.2 | 6 | time point |
| 60 | 95.6 | ± | 1.1 | 94.7 | ± | 1.0 | 6 | time point |
| 75 | 96.5 | ± | 0.3 | 95.6 | ± | 0.3 | 6 | time point |
| 90 | 97.1 | ± | 0.7 | 96.2 | ± | 0.7 | 6 | time point |

### Preparation: bulk-oralmix, Lot: a, Condition: syringe-25

| Days | Assay (mg/mL) | | | % of initial | | | n |  |
| --- | --- | --- | --- | --- | --- | --- | --- | --- |
| 0 | 101.0 | ± | 0.9 |  |  |  | 12 | time zero |
| 7 | 100.9 | ± | 1.1 | 100.0 | ± | 1.1 | 6 | time point |
| 14 | 95.4 | ± | 0.8 | 94.5 | ± | 0.8 | 6 | time point |
| 30 | 99.3 | ± | 0.5 | 98.3 | ± | 0.5 | 6 | time point |
| 45 | 97.4 | ± | 2.1 | 96.4 | ± | 2.1 | 6 | time point |
| 60 | 98.6 | ± | 1.4 | 97.7 | ± | 1.4 | 6 | time point |
| 75 | 96.5 | ± | 0.4 | 95.6 | ± | 0.4 | 6 | time point |
| 90 | 96.3 | ± | 2.1 | 95.3 | ± | 2.0 | 6 | time point |

### Preparation: tablet-oralmix, Lot: a, Condition: bottle-25

| Days | Assay (mg/mL) | | | % of initial | | | n |  |
| --- | --- | --- | --- | --- | --- | --- | --- | --- |
| 0 | 101.3 | ± | 1.9 |  |  |  | 12 | time zero |
| 7 | 96.7 | ± | 1.4 | 95.5 | ± | 1.3 | 6 | time point |
| 14 | 92.9 | ± | 1.3 | 91.7 | ± | 1.3 | 6 | time point |
| 30 | 100.2 | ± | 0.3 | 99.0 | ± | 0.3 | 6 | time point |
| 45 | 98.6 | ± | 0.8 | 97.4 | ± | 0.8 | 6 | time point |
| 60 | 96.7 | ± | 2.0 | 95.5 | ± | 2.0 | 6 | time point |
| 75 | 96.4 | ± | 0.9 | 95.2 | ± | 0.9 | 6 | time point |
| 90 | 94.3 | ± | 0.4 | 93.1 | ± | 0.4 | 6 | time point |

### Preparation: tablet-oralmix, Lot: a, Condition: syringe-25

| Days | Assay (mg/mL) | | | % of initial | | | n |  |
| --- | --- | --- | --- | --- | --- | --- | --- | --- |
| 0 | 101.3 | ± | 1.9 |  |  |  | 12 | time zero |
| 7 | 96.7 | ± | 2.6 | 95.5 | ± | 2.6 | 6 | time point |
| 14 | 93.3 | ± | 2.2 | 92.1 | ± | 2.1 | 6 | time point |
| 30 | 99.7 | ± | 0.8 | 98.5 | ± | 0.8 | 6 | time point |
| 45 | 96.7 | ± | 2.1 | 95.5 | ± | 2.0 | 6 | time point |
| 60 | 95.3 | ± | 1.1 | 94.1 | ± | 1.1 | 6 | time point |
| 75 | 97.1 | ± | 1.1 | 95.8 | ± | 1.1 | 6 | time point |
| 90 | 96.4 | ± | 1.8 | 95.2 | ± | 1.8 | 6 | time point |

### Preparation: bulk-oralmixsf, Lot: a, Condition: bottle-25

| Days | Assay (mg/mL) | | | % of initial | | | n |  |
| --- | --- | --- | --- | --- | --- | --- | --- | --- |
| 0 | 106.8 | ± | 0.9 |  |  |  | 12 | time zero |
| 7 | 107.7 | ± | 0.8 | 100.8 | ± | 0.8 | 6 | time point |
| 14 | 110.9 | ± | 2.6 | 103.8 | ± | 2.4 | 6 | time point |
| 30 | 111.5 | ± | 1.4 | 104.3 | ± | 1.3 | 6 | time point |
| 45 | 104.7 | ± | 1.3 | 98.0 | ± | 1.2 | 6 | time point |
| 60 | 108.4 | ± | 2.7 | 101.4 | ± | 2.6 | 6 | time point |
| 75 | 113.0 | ± | 4.0 | 105.7 | ± | 3.8 | 6 | time point |
| 90 | 110.7 | ± | 0.5 | 103.6 | ± | 0.5 | 6 | time point |

### Preparation: bulk-oralmixsf, Lot: a, Condition: syringe-25

| Days | Assay (mg/mL) | | | % of initial | | | n |  |
| --- | --- | --- | --- | --- | --- | --- | --- | --- |
| 0 | 106.8 | ± | 0.9 |  |  |  | 12 | time zero |
| 7 | 108.3 | ± | 1.5 | 101.4 | ± | 1.4 | 6 | time point |
| 14 | 109.6 | ± | 4.2 | 102.5 | ± | 3.9 | 6 | time point |
| 30 | 110.6 | ± | 1.1 | 103.5 | ± | 1.0 | 6 | time point |
| 45 | 106.9 | ± | 1.2 | 100.0 | ± | 1.2 | 6 | time point |
| 60 | 105.4 | ± | 0.4 | 98.6 | ± | 0.4 | 6 | time point |
| 75 | 109.9 | ± | 0.6 | 102.9 | ± | 0.5 | 6 | time point |
| 90 | 110.2 | ± | 1.1 | 103.1 | ± | 1.1 | 6 | time point |

### Preparation: tablet-oralmixsf, Lot: a, Condition: bottle-25

| Days | Assay (mg/mL) | | | % of initial | | | n |  |
| --- | --- | --- | --- | --- | --- | --- | --- | --- |
| 0 | 105.7 | ± | 0.8 |  |  |  | 12 | time zero |
| 7 | 105.9 | ± | 2.1 | 100.2 | ± | 2.0 | 6 | time point |
| 14 | 107.4 | ± | 2.8 | 101.6 | ± | 2.7 | 6 | time point |
| 30 | 112.2 | ± | 4.4 | 106.2 | ± | 4.1 | 6 | time point |
| 45 | 107.1 | ± | 0.9 | 101.4 | ± | 0.8 | 6 | time point |
| 60 | 105.2 | ± | 0.3 | 99.5 | ± | 0.3 | 6 | time point |
| 75 | 112.0 | ± | 0.3 | 105.9 | ± | 0.3 | 6 | time point |
| 90 | 110.7 | ± | 0.2 | 104.7 | ± | 0.2 | 6 | time point |

### Preparation: tablet-oralmixsf, Lot: a, Condition: syringe-25

| Days | Assay (mg/mL) | | | % of initial | | | n |  |
| --- | --- | --- | --- | --- | --- | --- | --- | --- |
| 0 | 105.7 | ± | 0.8 |  |  |  | 12 | time zero |
| 7 | 107.8 | ± | 0.7 | 102.0 | ± | 0.6 | 6 | time point |
| 14 | 112.8 | ± | 1.6 | 106.8 | ± | 1.5 | 6 | time point |
| 30 | 110.1 | ± | 1.9 | 104.2 | ± | 1.8 | 6 | time point |
| 45 | 105.7 | ± | 0.6 | 100.0 | ± | 0.5 | 6 | time point |
| 60 | 106.0 | ± | 0.3 | 100.3 | ± | 0.3 | 6 | time point |
| 75 | 110.0 | ± | 1.0 | 104.1 | ± | 1.0 | 6 | time point |
| 90 | 110.0 | ± | 1.6 | 104.1 | ± | 1.5 | 6 | time point |
