# Supplementary material for: Stability of gabapentin in extemporaneously compounded oral suspensions
Source: PLoS One. 2017 Apr 17;12(4):e0175208. doi: 10.1371/journal.pone.0175208 (PMC5393583; doi:10.1371/journal.pone.0175208)
Supplement: S2 Appendix — Archive containing the HPLC stability results as browsable html pages. (ZIP) [file pone.0175208.s003.zip › gaba_s2_html_results/gabapentin/index.html?preparation=bulk-oralmix&lot=a.html]

Stability Study Cruncher


### Preparation: bulk-oralmix, Lot: a

Assay: 101.0 ± 0.9 mg/mL (n = 12).

| Input String | Area | Cal Id | Cal Slope | Assay |  |
| --- | --- | --- | --- | --- | --- |
| gabapentin\_bulk-oralmix\_a\_bottle-25;1698762;;calt0om;time zero | 1698762 | calt0om | 16864 | 100.7 | calibration |
| gabapentin\_bulk-oralmix\_a\_bottle-25;1696193;;calt0om;time zero | 1696193 | calt0om | 16864 | 100.6 | calibration |
| gabapentin\_bulk-oralmix\_a\_bottle-25;1673690;;calt0om;time zero | 1673690 | calt0om | 16864 | 99.2 | calibration |
| gabapentin\_bulk-oralmix\_a\_bottle-25;1673184;;calt0om;time zero | 1673184 | calt0om | 16864 | 99.2 | calibration |
| gabapentin\_bulk-oralmix\_a\_bottle-25;1702967;;calt0om;time zero | 1702967 | calt0om | 16864 | 101.0 | calibration |
| gabapentin\_bulk-oralmix\_a\_bottle-25;1704488;;calt0om;time zero | 1704488 | calt0om | 16864 | 101.1 | calibration |
| gabapentin\_bulk-oralmix\_a\_syringe-25;1711793;;calt0om;time zero | 1711793 | calt0om | 16864 | 101.5 | calibration |
| gabapentin\_bulk-oralmix\_a\_syringe-25;1712943;;calt0om;time zero | 1712943 | calt0om | 16864 | 101.6 | calibration |
| gabapentin\_bulk-oralmix\_a\_syringe-25;1713003;;calt0om;time zero | 1713003 | calt0om | 16864 | 101.6 | calibration |
| gabapentin\_bulk-oralmix\_a\_syringe-25;1707200;;calt0om;time zero | 1707200 | calt0om | 16864 | 101.2 | calibration |
| gabapentin\_bulk-oralmix\_a\_syringe-25;1719451;;calt0om;time zero | 1719451 | calt0om | 16864 | 102.0 | calibration |
| gabapentin\_bulk-oralmix\_a\_syringe-25;1718452;;calt0om;time zero | 1718452 | calt0om | 16864 | 101.9 | calibration |
