# Supplementary material for: Stability of gabapentin in extemporaneously compounded oral suspensions
Source: PLoS One. 2017 Apr 17;12(4):e0175208. doi: 10.1371/journal.pone.0175208 (PMC5393583; doi:10.1371/journal.pone.0175208)
Supplement: S2 Appendix — Archive containing the HPLC stability results as browsable html pages. (ZIP) [file pone.0175208.s003.zip › gaba_s2_html_results/gabapentin/index.html?preparation=bulk-oralmix&lot=a&condition=bottle-25&time=14.html]

Stability Study Cruncher


### Preparation: bulk-oralmix, Lot: a, Condition: bottle-25, Time: 14

Assay (mg/mL): 95.6 ± 2.0 (n = 6);
Assay (%TZ): 94.7 ± 2.0 (n = 6).

| Input String | Area | Cal Id | Cal Slope | Assay | Assay TZ | Assay %TZ |  |
| --- | --- | --- | --- | --- | --- | --- | --- |
| gabapentin\_bulk-oralmix\_a\_bottle-25\_14;1610756;;calt0om;stability | 1610756 | calt0om | 16864 | 95.5 | 101.0 | 94.6 | calibration, time zero |
| gabapentin\_bulk-oralmix\_a\_bottle-25\_14;1606759;;calt0om;stability | 1606759 | calt0om | 16864 | 95.3 | 101.0 | 94.4 | calibration, time zero |
| gabapentin\_bulk-oralmix\_a\_bottle-25\_14;1652724;;calt0om;stability | 1652724 | calt0om | 16864 | 98.0 | 101.0 | 97.1 | calibration, time zero |
| gabapentin\_bulk-oralmix\_a\_bottle-25\_14;1648938;;calt0om;stability | 1648938 | calt0om | 16864 | 97.8 | 101.0 | 96.8 | calibration, time zero |
| gabapentin\_bulk-oralmix\_a\_bottle-25\_14;1576783;;calt0om;stability | 1576783 | calt0om | 16864 | 93.5 | 101.0 | 92.6 | calibration, time zero |
| gabapentin\_bulk-oralmix\_a\_bottle-25\_14;1575339;;calt0om;stability | 1575339 | calt0om | 16864 | 93.4 | 101.0 | 92.5 | calibration, time zero |
