# Supplementary material for: Stability of gabapentin in extemporaneously compounded oral suspensions
Source: PLoS One. 2017 Apr 17;12(4):e0175208. doi: 10.1371/journal.pone.0175208 (PMC5393583; doi:10.1371/journal.pone.0175208)
Supplement: S2 Appendix — Archive containing the HPLC stability results as browsable html pages. (ZIP) [file pone.0175208.s003.zip › gaba_s2_html_results/gabapentin/index.html?preparation=bulk-oralmix&lot=a&condition=bottle-25&time=30.html]

Stability Study Cruncher


### Preparation: bulk-oralmix, Lot: a, Condition: bottle-25, Time: 30

Assay (mg/mL): 99.2 ± 0.6 (n = 6);
Assay (%TZ): 98.3 ± 0.6 (n = 6).

| Input String | Area | Cal Id | Cal Slope | Assay | Assay TZ | Assay %TZ |  |
| --- | --- | --- | --- | --- | --- | --- | --- |
| gabapentin\_bulk-oralmix\_a\_bottle-25\_30;1676075;;calt0om;stability | 1676075 | calt0om | 16864 | 99.4 | 101.0 | 98.4 | calibration, time zero |
| gabapentin\_bulk-oralmix\_a\_bottle-25\_30;1677174;;calt0om;stability | 1677174 | calt0om | 16864 | 99.5 | 101.0 | 98.5 | calibration, time zero |
| gabapentin\_bulk-oralmix\_a\_bottle-25\_30;1659379;;calt0om;stability | 1659379 | calt0om | 16864 | 98.4 | 101.0 | 97.5 | calibration, time zero |
| gabapentin\_bulk-oralmix\_a\_bottle-25\_30;1662572;;calt0om;stability | 1662572 | calt0om | 16864 | 98.6 | 101.0 | 97.6 | calibration, time zero |
| gabapentin\_bulk-oralmix\_a\_bottle-25\_30;1680687;;calt0om;stability | 1680687 | calt0om | 16864 | 99.7 | 101.0 | 98.7 | calibration, time zero |
| gabapentin\_bulk-oralmix\_a\_bottle-25\_30;1682960;;calt0om;stability | 1682960 | calt0om | 16864 | 99.8 | 101.0 | 98.8 | calibration, time zero |
