# Supplementary material for: Stability of gabapentin in extemporaneously compounded oral suspensions
Source: PLoS One. 2017 Apr 17;12(4):e0175208. doi: 10.1371/journal.pone.0175208 (PMC5393583; doi:10.1371/journal.pone.0175208)
Supplement: S2 Appendix — Archive containing the HPLC stability results as browsable html pages. (ZIP) [file pone.0175208.s003.zip › gaba_s2_html_results/gabapentin/index.html?preparation=bulk-oralmix&lot=a&condition=bottle-25&time=45.html]

Stability Study Cruncher


### Preparation: bulk-oralmix, Lot: a, Condition: bottle-25, Time: 45

Assay (mg/mL): 97.8 ± 3.2 (n = 6);
Assay (%TZ): 96.9 ± 3.2 (n = 6).

| Input String | Area | Cal Id | Cal Slope | Assay | Assay TZ | Assay %TZ |  |
| --- | --- | --- | --- | --- | --- | --- | --- |
| gabapentin\_bulk-oralmix\_a\_bottle-25\_45;1664879;;calt0om;stability | 1664879 | calt0om | 16864 | 98.7 | 101.0 | 97.8 | calibration, time zero |
| gabapentin\_bulk-oralmix\_a\_bottle-25\_45;1686072;;calt0om;stability | 1686072 | calt0om | 16864 | 100.0 | 101.0 | 99.0 | calibration, time zero |
| gabapentin\_bulk-oralmix\_a\_bottle-25\_45;1580857;;calt0om;stability | 1580857 | calt0om | 16864 | 93.7 | 101.0 | 92.8 | calibration, time zero |
| gabapentin\_bulk-oralmix\_a\_bottle-25\_45;1580954;;calt0om;stability | 1580954 | calt0om | 16864 | 93.7 | 101.0 | 92.9 | calibration, time zero |
| gabapentin\_bulk-oralmix\_a\_bottle-25\_45;1690361;;calt0om;stability | 1690361 | calt0om | 16864 | 100.2 | 101.0 | 99.3 | calibration, time zero |
| gabapentin\_bulk-oralmix\_a\_bottle-25\_45;1697519;;calt0om;stability | 1697519 | calt0om | 16864 | 100.7 | 101.0 | 99.7 | calibration, time zero |
