# Supplementary material for: Stability of gabapentin in extemporaneously compounded oral suspensions
Source: PLoS One. 2017 Apr 17;12(4):e0175208. doi: 10.1371/journal.pone.0175208 (PMC5393583; doi:10.1371/journal.pone.0175208)
Supplement: S2 Appendix — Archive containing the HPLC stability results as browsable html pages. (ZIP) [file pone.0175208.s003.zip › gaba_s2_html_results/gabapentin/index.html?preparation=bulk-oralmix&lot=a&condition=bottle-25&time=60.html]

Stability Study Cruncher


### Preparation: bulk-oralmix, Lot: a, Condition: bottle-25, Time: 60

Assay (mg/mL): 95.6 ± 1.1 (n = 6);
Assay (%TZ): 94.7 ± 1.0 (n = 6).

| Input String | Area | Cal Id | Cal Slope | Assay | Assay TZ | Assay %TZ |  |
| --- | --- | --- | --- | --- | --- | --- | --- |
| gabapentin\_bulk-oralmix\_a\_bottle-25\_60;1599503;;calt0om;stability | 1599503 | calt0om | 16864 | 94.8 | 101.0 | 93.9 | calibration, time zero |
| gabapentin\_bulk-oralmix\_a\_bottle-25\_60;1617430;;calt0om;stability | 1617430 | calt0om | 16864 | 95.9 | 101.0 | 95.0 | calibration, time zero |
| gabapentin\_bulk-oralmix\_a\_bottle-25\_60;1635396;;calt0om;stability | 1635396 | calt0om | 16864 | 97.0 | 101.0 | 96.0 | calibration, time zero |
| gabapentin\_bulk-oralmix\_a\_bottle-25\_60;1630616;;calt0om;stability | 1630616 | calt0om | 16864 | 96.7 | 101.0 | 95.8 | calibration, time zero |
| gabapentin\_bulk-oralmix\_a\_bottle-25\_60;1596847;;calt0om;stability | 1596847 | calt0om | 16864 | 94.7 | 101.0 | 93.8 | calibration, time zero |
| gabapentin\_bulk-oralmix\_a\_bottle-25\_60;1595313;;calt0om;stability | 1595313 | calt0om | 16864 | 94.6 | 101.0 | 93.7 | calibration, time zero |
