# Supplementary material for: Stability of gabapentin in extemporaneously compounded oral suspensions
Source: PLoS One. 2017 Apr 17;12(4):e0175208. doi: 10.1371/journal.pone.0175208 (PMC5393583; doi:10.1371/journal.pone.0175208)
Supplement: S2 Appendix — Archive containing the HPLC stability results as browsable html pages. (ZIP) [file pone.0175208.s003.zip › gaba_s2_html_results/gabapentin/index.html?preparation=bulk-oralmix&lot=a&condition=bottle-25&time=75.html]

Stability Study Cruncher


### Preparation: bulk-oralmix, Lot: a, Condition: bottle-25, Time: 75

Assay (mg/mL): 96.5 ± 0.3 (n = 6);
Assay (%TZ): 95.6 ± 0.3 (n = 6).

| Input String | Area | Cal Id | Cal Slope | Assay | Assay TZ | Assay %TZ |  |
| --- | --- | --- | --- | --- | --- | --- | --- |
| gabapentin\_bulk-oralmix\_a\_bottle-25\_75;1629426;;calt0om;stability | 1629426 | calt0om | 16864 | 96.6 | 101.0 | 95.7 | calibration, time zero |
| gabapentin\_bulk-oralmix\_a\_bottle-25\_75;1627941;;calt0om;stability | 1627941 | calt0om | 16864 | 96.5 | 101.0 | 95.6 | calibration, time zero |
| gabapentin\_bulk-oralmix\_a\_bottle-25\_75;1635930;;calt0om;stability | 1635930 | calt0om | 16864 | 97.0 | 101.0 | 96.1 | calibration, time zero |
| gabapentin\_bulk-oralmix\_a\_bottle-25\_75;1622113;;calt0om;stability | 1622113 | calt0om | 16864 | 96.2 | 101.0 | 95.3 | calibration, time zero |
| gabapentin\_bulk-oralmix\_a\_bottle-25\_75;1624841;;calt0om;stability | 1624841 | calt0om | 16864 | 96.3 | 101.0 | 95.4 | calibration, time zero |
| gabapentin\_bulk-oralmix\_a\_bottle-25\_75;1623621;;calt0om;stability | 1623621 | calt0om | 16864 | 96.3 | 101.0 | 95.4 | calibration, time zero |
