# Supplementary material for: Stability of gabapentin in extemporaneously compounded oral suspensions
Source: PLoS One. 2017 Apr 17;12(4):e0175208. doi: 10.1371/journal.pone.0175208 (PMC5393583; doi:10.1371/journal.pone.0175208)
Supplement: S2 Appendix — Archive containing the HPLC stability results as browsable html pages. (ZIP) [file pone.0175208.s003.zip › gaba_s2_html_results/gabapentin/index.html?preparation=bulk-oralmix&lot=a&condition=bottle-25&time=90.html]

Stability Study Cruncher


### Preparation: bulk-oralmix, Lot: a, Condition: bottle-25, Time: 90

Assay (mg/mL): 97.1 ± 0.7 (n = 6);
Assay (%TZ): 96.2 ± 0.7 (n = 6).

| Input String | Area | Cal Id | Cal Slope | Assay | Assay TZ | Assay %TZ |  |
| --- | --- | --- | --- | --- | --- | --- | --- |
| gabapentin\_bulk-oralmix\_a\_bottle-25\_90;1647534;;calt0om;stability | 1647534 | calt0om | 16864 | 97.7 | 101.0 | 96.8 | calibration, time zero |
| gabapentin\_bulk-oralmix\_a\_bottle-25\_90;1638351;;calt0om;stability | 1638351 | calt0om | 16864 | 97.2 | 101.0 | 96.2 | calibration, time zero |
| gabapentin\_bulk-oralmix\_a\_bottle-25\_90;1624674;;calt0om;stability | 1624674 | calt0om | 16864 | 96.3 | 101.0 | 95.4 | calibration, time zero |
| gabapentin\_bulk-oralmix\_a\_bottle-25\_90;1622749;;calt0om;stability | 1622749 | calt0om | 16864 | 96.2 | 101.0 | 95.3 | calibration, time zero |
| gabapentin\_bulk-oralmix\_a\_bottle-25\_90;1648771;;calt0om;stability | 1648771 | calt0om | 16864 | 97.8 | 101.0 | 96.8 | calibration, time zero |
| gabapentin\_bulk-oralmix\_a\_bottle-25\_90;1646402;;calt0om;stability | 1646402 | calt0om | 16864 | 97.6 | 101.0 | 96.7 | calibration, time zero |
