# Supplementary material for: Stability of gabapentin in extemporaneously compounded oral suspensions
Source: PLoS One. 2017 Apr 17;12(4):e0175208. doi: 10.1371/journal.pone.0175208 (PMC5393583; doi:10.1371/journal.pone.0175208)
Supplement: S2 Appendix — Archive containing the HPLC stability results as browsable html pages. (ZIP) [file pone.0175208.s003.zip › gaba_s2_html_results/gabapentin/index.html?preparation=bulk-oralmix&lot=a&condition=syringe-25&time=7.html]

Stability Study Cruncher


### Preparation: bulk-oralmix, Lot: a, Condition: syringe-25, Time: 7

Assay (mg/mL): 100.9 ± 1.1 (n = 6);
Assay (%TZ): 100.0 ± 1.1 (n = 6).

| Input String | Area | Cal Id | Cal Slope | Assay | Assay TZ | Assay %TZ |  |
| --- | --- | --- | --- | --- | --- | --- | --- |
| gabapentin\_bulk-oralmix\_a\_syringe-25\_7;1703942;;calt0om;stability | 1703942 | calt0om | 16864 | 101.0 | 101.0 | 100.1 | calibration, time zero |
| gabapentin\_bulk-oralmix\_a\_syringe-25\_7;1716196;;calt0om;stability | 1716196 | calt0om | 16864 | 101.8 | 101.0 | 100.8 | calibration, time zero |
| gabapentin\_bulk-oralmix\_a\_syringe-25\_7;1714405;;calt0om;stability | 1714405 | calt0om | 16864 | 101.7 | 101.0 | 100.7 | calibration, time zero |
| gabapentin\_bulk-oralmix\_a\_syringe-25\_7;1719191;;calt0om;stability | 1719191 | calt0om | 16864 | 101.9 | 101.0 | 101.0 | calibration, time zero |
| gabapentin\_bulk-oralmix\_a\_syringe-25\_7;1680359;;calt0om;stability | 1680359 | calt0om | 16864 | 99.6 | 101.0 | 98.7 | calibration, time zero |
| gabapentin\_bulk-oralmix\_a\_syringe-25\_7;1679061;;calt0om;stability | 1679061 | calt0om | 16864 | 99.6 | 101.0 | 98.6 | calibration, time zero |
