# Supplementary material for: Stability of gabapentin in extemporaneously compounded oral suspensions
Source: PLoS One. 2017 Apr 17;12(4):e0175208. doi: 10.1371/journal.pone.0175208 (PMC5393583; doi:10.1371/journal.pone.0175208)
Supplement: S2 Appendix — Archive containing the HPLC stability results as browsable html pages. (ZIP) [file pone.0175208.s003.zip › gaba_s2_html_results/gabapentin/index.html?preparation=bulk-oralmix&lot=a&condition=syringe-25&time=14.html]

Stability Study Cruncher


### Preparation: bulk-oralmix, Lot: a, Condition: syringe-25, Time: 14

Assay (mg/mL): 95.4 ± 0.8 (n = 6);
Assay (%TZ): 94.5 ± 0.8 (n = 6).

| Input String | Area | Cal Id | Cal Slope | Assay | Assay TZ | Assay %TZ |  |
| --- | --- | --- | --- | --- | --- | --- | --- |
| gabapentin\_bulk-oralmix\_a\_syringe-25\_14;1599529;;calt0om;stability | 1599529 | calt0om | 16864 | 94.8 | 101.0 | 93.9 | calibration, time zero |
| gabapentin\_bulk-oralmix\_a\_syringe-25\_14;1595895;;calt0om;stability | 1595895 | calt0om | 16864 | 94.6 | 101.0 | 93.7 | calibration, time zero |
| gabapentin\_bulk-oralmix\_a\_syringe-25\_14;1629943;;calt0om;stability | 1629943 | calt0om | 16864 | 96.7 | 101.0 | 95.7 | calibration, time zero |
| gabapentin\_bulk-oralmix\_a\_syringe-25\_14;1606978;;calt0om;stability | 1606978 | calt0om | 16864 | 95.3 | 101.0 | 94.4 | calibration, time zero |
| gabapentin\_bulk-oralmix\_a\_syringe-25\_14;1620061;;calt0om;stability | 1620061 | calt0om | 16864 | 96.1 | 101.0 | 95.1 | calibration, time zero |
| gabapentin\_bulk-oralmix\_a\_syringe-25\_14;1604836;;calt0om;stability | 1604836 | calt0om | 16864 | 95.2 | 101.0 | 94.3 | calibration, time zero |
