# Supplementary material for: Stability of gabapentin in extemporaneously compounded oral suspensions
Source: PLoS One. 2017 Apr 17;12(4):e0175208. doi: 10.1371/journal.pone.0175208 (PMC5393583; doi:10.1371/journal.pone.0175208)
Supplement: S2 Appendix — Archive containing the HPLC stability results as browsable html pages. (ZIP) [file pone.0175208.s003.zip › gaba_s2_html_results/gabapentin/index.html?preparation=bulk-oralmix&lot=a&condition=syringe-25&time=30.html]

Stability Study Cruncher


### Preparation: bulk-oralmix, Lot: a, Condition: syringe-25, Time: 30

Assay (mg/mL): 99.3 ± 0.5 (n = 6);
Assay (%TZ): 98.3 ± 0.5 (n = 6).

| Input String | Area | Cal Id | Cal Slope | Assay | Assay TZ | Assay %TZ |  |
| --- | --- | --- | --- | --- | --- | --- | --- |
| gabapentin\_bulk-oralmix\_a\_syringe-25\_30;1667931;;calt0om;stability | 1667931 | calt0om | 16864 | 98.9 | 101.0 | 98.0 | calibration, time zero |
| gabapentin\_bulk-oralmix\_a\_syringe-25\_30;1663553;;calt0om;stability | 1663553 | calt0om | 16864 | 98.6 | 101.0 | 97.7 | calibration, time zero |
| gabapentin\_bulk-oralmix\_a\_syringe-25\_30;1673294;;calt0om;stability | 1673294 | calt0om | 16864 | 99.2 | 101.0 | 98.3 | calibration, time zero |
| gabapentin\_bulk-oralmix\_a\_syringe-25\_30;1675891;;calt0om;stability | 1675891 | calt0om | 16864 | 99.4 | 101.0 | 98.4 | calibration, time zero |
| gabapentin\_bulk-oralmix\_a\_syringe-25\_30;1683439;;calt0om;stability | 1683439 | calt0om | 16864 | 99.8 | 101.0 | 98.9 | calibration, time zero |
| gabapentin\_bulk-oralmix\_a\_syringe-25\_30;1682590;;calt0om;stability | 1682590 | calt0om | 16864 | 99.8 | 101.0 | 98.8 | calibration, time zero |
