# Supplementary material for: Stability of gabapentin in extemporaneously compounded oral suspensions
Source: PLoS One. 2017 Apr 17;12(4):e0175208. doi: 10.1371/journal.pone.0175208 (PMC5393583; doi:10.1371/journal.pone.0175208)
Supplement: S2 Appendix — Archive containing the HPLC stability results as browsable html pages. (ZIP) [file pone.0175208.s003.zip › gaba_s2_html_results/gabapentin/index.html?preparation=bulk-oralmix&lot=a&condition=syringe-25&time=60.html]

Stability Study Cruncher


### Preparation: bulk-oralmix, Lot: a, Condition: syringe-25, Time: 60

Assay (mg/mL): 98.6 ± 1.4 (n = 6);
Assay (%TZ): 97.7 ± 1.4 (n = 6).

| Input String | Area | Cal Id | Cal Slope | Assay | Assay TZ | Assay %TZ |  |
| --- | --- | --- | --- | --- | --- | --- | --- |
| gabapentin\_bulk-oralmix\_a\_syringe-25\_60;1689016;;calt0om;stability | 1689016 | calt0om | 16864 | 100.2 | 101.0 | 99.2 | calibration, time zero |
| gabapentin\_bulk-oralmix\_a\_syringe-25\_60;1687392;;calt0om;stability | 1687392 | calt0om | 16864 | 100.1 | 101.0 | 99.1 | calibration, time zero |
| gabapentin\_bulk-oralmix\_a\_syringe-25\_60;1645715;;calt0om;stability | 1645715 | calt0om | 16864 | 97.6 | 101.0 | 96.7 | calibration, time zero |
| gabapentin\_bulk-oralmix\_a\_syringe-25\_60;1678892;;calt0om;stability | 1678892 | calt0om | 16864 | 99.6 | 101.0 | 98.6 | calibration, time zero |
| gabapentin\_bulk-oralmix\_a\_syringe-25\_60;1637849;;calt0om;stability | 1637849 | calt0om | 16864 | 97.1 | 101.0 | 96.2 | calibration, time zero |
| gabapentin\_bulk-oralmix\_a\_syringe-25\_60;1642206;;calt0om;stability | 1642206 | calt0om | 16864 | 97.4 | 101.0 | 96.4 | calibration, time zero |
