# Supplementary material for: Stability of gabapentin in extemporaneously compounded oral suspensions
Source: PLoS One. 2017 Apr 17;12(4):e0175208. doi: 10.1371/journal.pone.0175208 (PMC5393583; doi:10.1371/journal.pone.0175208)
Supplement: S2 Appendix — Archive containing the HPLC stability results as browsable html pages. (ZIP) [file pone.0175208.s003.zip › gaba_s2_html_results/gabapentin/index.html?preparation=bulk-oralmix&lot=a&condition=syringe-25&time=75.html]

Stability Study Cruncher


### Preparation: bulk-oralmix, Lot: a, Condition: syringe-25, Time: 75

Assay (mg/mL): 96.5 ± 0.4 (n = 6);
Assay (%TZ): 95.6 ± 0.4 (n = 6).

| Input String | Area | Cal Id | Cal Slope | Assay | Assay TZ | Assay %TZ |  |
| --- | --- | --- | --- | --- | --- | --- | --- |
| gabapentin\_bulk-oralmix\_a\_syringe-25\_75;1625904;;calt0om;stability | 1625904 | calt0om | 16864 | 96.4 | 101.0 | 95.5 | calibration, time zero |
| gabapentin\_bulk-oralmix\_a\_syringe-25\_75;1631195;;calt0om;stability | 1631195 | calt0om | 16864 | 96.7 | 101.0 | 95.8 | calibration, time zero |
| gabapentin\_bulk-oralmix\_a\_syringe-25\_75;1630224;;calt0om;stability | 1630224 | calt0om | 16864 | 96.7 | 101.0 | 95.7 | calibration, time zero |
| gabapentin\_bulk-oralmix\_a\_syringe-25\_75;1636310;;calt0om;stability | 1636310 | calt0om | 16864 | 97.0 | 101.0 | 96.1 | calibration, time zero |
| gabapentin\_bulk-oralmix\_a\_syringe-25\_75;1619407;;calt0om;stability | 1619407 | calt0om | 16864 | 96.0 | 101.0 | 95.1 | calibration, time zero |
| gabapentin\_bulk-oralmix\_a\_syringe-25\_75;1619754;;calt0om;stability | 1619754 | calt0om | 16864 | 96.0 | 101.0 | 95.1 | calibration, time zero |
