# Supplementary material for: Stability of gabapentin in extemporaneously compounded oral suspensions
Source: PLoS One. 2017 Apr 17;12(4):e0175208. doi: 10.1371/journal.pone.0175208 (PMC5393583; doi:10.1371/journal.pone.0175208)
Supplement: S2 Appendix — Archive containing the HPLC stability results as browsable html pages. (ZIP) [file pone.0175208.s003.zip › gaba_s2_html_results/gabapentin/index.html?preparation=bulk-oralmix&lot=a&condition=syringe-25&time=90.html]

Stability Study Cruncher


### Preparation: bulk-oralmix, Lot: a, Condition: syringe-25, Time: 90

Assay (mg/mL): 96.3 ± 2.1 (n = 6);
Assay (%TZ): 95.3 ± 2.0 (n = 6).

| Input String | Area | Cal Id | Cal Slope | Assay | Assay TZ | Assay %TZ |  |
| --- | --- | --- | --- | --- | --- | --- | --- |
| gabapentin\_bulk-oralmix\_a\_syringe-25\_90;1616202;;calt0om;stability | 1616202 | calt0om | 16864 | 95.8 | 101.0 | 94.9 | calibration, time zero |
| gabapentin\_bulk-oralmix\_a\_syringe-25\_90;1621015;;calt0om;stability | 1621015 | calt0om | 16864 | 96.1 | 101.0 | 95.2 | calibration, time zero |
| gabapentin\_bulk-oralmix\_a\_syringe-25\_90;1668518;;calt0om;stability | 1668518 | calt0om | 16864 | 98.9 | 101.0 | 98.0 | calibration, time zero |
| gabapentin\_bulk-oralmix\_a\_syringe-25\_90;1659355;;calt0om;stability | 1659355 | calt0om | 16864 | 98.4 | 101.0 | 97.5 | calibration, time zero |
| gabapentin\_bulk-oralmix\_a\_syringe-25\_90;1592633;;calt0om;stability | 1592633 | calt0om | 16864 | 94.4 | 101.0 | 93.5 | calibration, time zero |
| gabapentin\_bulk-oralmix\_a\_syringe-25\_90;1581711;;calt0om;stability | 1581711 | calt0om | 16864 | 93.8 | 101.0 | 92.9 | calibration, time zero |
