# Supplementary material for: Stability of gabapentin in extemporaneously compounded oral suspensions
Source: PLoS One. 2017 Apr 17;12(4):e0175208. doi: 10.1371/journal.pone.0175208 (PMC5393583; doi:10.1371/journal.pone.0175208)
Supplement: S2 Appendix — Archive containing the HPLC stability results as browsable html pages. (ZIP) [file pone.0175208.s003.zip › gaba_s2_html_results/gabapentin/index.html?preparation=tablet-oralmix&lot=a&condition=bottle-25&time=7.html]

Stability Study Cruncher


### Preparation: tablet-oralmix, Lot: a, Condition: bottle-25, Time: 7

Assay (mg/mL): 96.7 ± 1.4 (n = 6);
Assay (%TZ): 95.5 ± 1.3 (n = 6).

| Input String | Area | Cal Id | Cal Slope | Assay | Assay TZ | Assay %TZ |  |
| --- | --- | --- | --- | --- | --- | --- | --- |
| gabapentin\_tablet-oralmix\_a\_bottle-25\_7;1647571;;calt0om;stability | 1647571 | calt0om | 16864 | 97.7 | 101.3 | 96.5 | calibration, time zero |
| gabapentin\_tablet-oralmix\_a\_bottle-25\_7;1667511;;calt0om;stability | 1667511 | calt0om | 16864 | 98.9 | 101.3 | 97.6 | calibration, time zero |
| gabapentin\_tablet-oralmix\_a\_bottle-25\_7;1615253;;calt0om;stability | 1615253 | calt0om | 16864 | 95.8 | 101.3 | 94.6 | calibration, time zero |
| gabapentin\_tablet-oralmix\_a\_bottle-25\_7;1624835;;calt0om;stability | 1624835 | calt0om | 16864 | 96.3 | 101.3 | 95.1 | calibration, time zero |
| gabapentin\_tablet-oralmix\_a\_bottle-25\_7;1607228;;calt0om;stability | 1607228 | calt0om | 16864 | 95.3 | 101.3 | 94.1 | calibration, time zero |
| gabapentin\_tablet-oralmix\_a\_bottle-25\_7;1617723;;calt0om;stability | 1617723 | calt0om | 16864 | 95.9 | 101.3 | 94.7 | calibration, time zero |
