# Supplementary material for: Stability of gabapentin in extemporaneously compounded oral suspensions
Source: PLoS One. 2017 Apr 17;12(4):e0175208. doi: 10.1371/journal.pone.0175208 (PMC5393583; doi:10.1371/journal.pone.0175208)
Supplement: S2 Appendix — Archive containing the HPLC stability results as browsable html pages. (ZIP) [file pone.0175208.s003.zip › gaba_s2_html_results/gabapentin/index.html?preparation=tablet-oralmix&lot=a&condition=bottle-25&time=14.html]

Stability Study Cruncher


### Preparation: tablet-oralmix, Lot: a, Condition: bottle-25, Time: 14

Assay (mg/mL): 92.9 ± 1.3 (n = 6);
Assay (%TZ): 91.7 ± 1.3 (n = 6).

| Input String | Area | Cal Id | Cal Slope | Assay | Assay TZ | Assay %TZ |  |
| --- | --- | --- | --- | --- | --- | --- | --- |
| gabapentin\_tablet-oralmix\_a\_bottle-25\_14;1595021;;calt0om;stability | 1595021 | calt0om | 16864 | 94.6 | 101.3 | 93.4 | calibration, time zero |
| gabapentin\_tablet-oralmix\_a\_bottle-25\_14;1592351;;calt0om;stability | 1592351 | calt0om | 16864 | 94.4 | 101.3 | 93.2 | calibration, time zero |
| gabapentin\_tablet-oralmix\_a\_bottle-25\_14;1556468;;calt0om;stability | 1556468 | calt0om | 16864 | 92.3 | 101.3 | 91.1 | calibration, time zero |
| gabapentin\_tablet-oralmix\_a\_bottle-25\_14;1556728;;calt0om;stability | 1556728 | calt0om | 16864 | 92.3 | 101.3 | 91.2 | calibration, time zero |
| gabapentin\_tablet-oralmix\_a\_bottle-25\_14;1542714;;calt0om;stability | 1542714 | calt0om | 16864 | 91.5 | 101.3 | 90.3 | calibration, time zero |
| gabapentin\_tablet-oralmix\_a\_bottle-25\_14;1553224;;calt0om;stability | 1553224 | calt0om | 16864 | 92.1 | 101.3 | 91.0 | calibration, time zero |
