# Supplementary material for: Stability of gabapentin in extemporaneously compounded oral suspensions
Source: PLoS One. 2017 Apr 17;12(4):e0175208. doi: 10.1371/journal.pone.0175208 (PMC5393583; doi:10.1371/journal.pone.0175208)
Supplement: S2 Appendix — Archive containing the HPLC stability results as browsable html pages. (ZIP) [file pone.0175208.s003.zip › gaba_s2_html_results/gabapentin/index.html?preparation=tablet-oralmix&lot=a&condition=syringe-25&time=14.html]

Stability Study Cruncher


### Preparation: tablet-oralmix, Lot: a, Condition: syringe-25, Time: 14

Assay (mg/mL): 93.3 ± 2.2 (n = 6);
Assay (%TZ): 92.1 ± 2.1 (n = 6).

| Input String | Area | Cal Id | Cal Slope | Assay | Assay TZ | Assay %TZ |  |
| --- | --- | --- | --- | --- | --- | --- | --- |
| gabapentin\_tablet-oralmix\_a\_syringe-25\_14;1559213;;calt0om;stability | 1559213 | calt0om | 16864 | 92.5 | 101.3 | 91.3 | calibration, time zero |
| gabapentin\_tablet-oralmix\_a\_syringe-25\_14;1559206;;calt0om;stability | 1559206 | calt0om | 16864 | 92.5 | 101.3 | 91.3 | calibration, time zero |
| gabapentin\_tablet-oralmix\_a\_syringe-25\_14;1545870;;calt0om;stability | 1545870 | calt0om | 16864 | 91.7 | 101.3 | 90.5 | calibration, time zero |
| gabapentin\_tablet-oralmix\_a\_syringe-25\_14;1538724;;calt0om;stability | 1538724 | calt0om | 16864 | 91.2 | 101.3 | 90.1 | calibration, time zero |
| gabapentin\_tablet-oralmix\_a\_syringe-25\_14;1612215;;calt0om;stability | 1612215 | calt0om | 16864 | 95.6 | 101.3 | 94.4 | calibration, time zero |
| gabapentin\_tablet-oralmix\_a\_syringe-25\_14;1626189;;calt0om;stability | 1626189 | calt0om | 16864 | 96.4 | 101.3 | 95.2 | calibration, time zero |
