# Supplementary material for: Stability of gabapentin in extemporaneously compounded oral suspensions
Source: PLoS One. 2017 Apr 17;12(4):e0175208. doi: 10.1371/journal.pone.0175208 (PMC5393583; doi:10.1371/journal.pone.0175208)
Supplement: S2 Appendix — Archive containing the HPLC stability results as browsable html pages. (ZIP) [file pone.0175208.s003.zip › gaba_s2_html_results/gabapentin/index.html?preparation=tablet-oralmix&lot=a&condition=bottle-25&time=30.html]

Stability Study Cruncher


### Preparation: tablet-oralmix, Lot: a, Condition: bottle-25, Time: 30

Assay (mg/mL): 100.2 ± 0.3 (n = 6);
Assay (%TZ): 99.0 ± 0.3 (n = 6).

| Input String | Area | Cal Id | Cal Slope | Assay | Assay TZ | Assay %TZ |  |
| --- | --- | --- | --- | --- | --- | --- | --- |
| gabapentin\_tablet-oralmix\_a\_bottle-25\_30;1694532;;calt0om;stability | 1694532 | calt0om | 16864 | 100.5 | 101.3 | 99.2 | calibration, time zero |
| gabapentin\_tablet-oralmix\_a\_bottle-25\_30;1696271;;calt0om;stability | 1696271 | calt0om | 16864 | 100.6 | 101.3 | 99.3 | calibration, time zero |
| gabapentin\_tablet-oralmix\_a\_bottle-25\_30;1690080;;calt0om;stability | 1690080 | calt0om | 16864 | 100.2 | 101.3 | 99.0 | calibration, time zero |
| gabapentin\_tablet-oralmix\_a\_bottle-25\_30;1691398;;calt0om;stability | 1691398 | calt0om | 16864 | 100.3 | 101.3 | 99.0 | calibration, time zero |
| gabapentin\_tablet-oralmix\_a\_bottle-25\_30;1682976;;calt0om;stability | 1682976 | calt0om | 16864 | 99.8 | 101.3 | 98.6 | calibration, time zero |
| gabapentin\_tablet-oralmix\_a\_bottle-25\_30;1686941;;calt0om;stability | 1686941 | calt0om | 16864 | 100.0 | 101.3 | 98.8 | calibration, time zero |
