# Supplementary material for: Stability of gabapentin in extemporaneously compounded oral suspensions
Source: PLoS One. 2017 Apr 17;12(4):e0175208. doi: 10.1371/journal.pone.0175208 (PMC5393583; doi:10.1371/journal.pone.0175208)
Supplement: S2 Appendix — Archive containing the HPLC stability results as browsable html pages. (ZIP) [file pone.0175208.s003.zip › gaba_s2_html_results/gabapentin/index.html?preparation=tablet-oralmix&lot=a&condition=bottle-25&time=45.html]

Stability Study Cruncher


### Preparation: tablet-oralmix, Lot: a, Condition: bottle-25, Time: 45

Assay (mg/mL): 98.6 ± 0.8 (n = 6);
Assay (%TZ): 97.4 ± 0.8 (n = 6).

| Input String | Area | Cal Id | Cal Slope | Assay | Assay TZ | Assay %TZ |  |
| --- | --- | --- | --- | --- | --- | --- | --- |
| gabapentin\_tablet-oralmix\_a\_bottle-25\_45;1646819;;calt0om;stability | 1646819 | calt0om | 16864 | 97.7 | 101.3 | 96.4 | calibration, time zero |
| gabapentin\_tablet-oralmix\_a\_bottle-25\_45;1647498;;calt0om;stability | 1647498 | calt0om | 16864 | 97.7 | 101.3 | 96.5 | calibration, time zero |
| gabapentin\_tablet-oralmix\_a\_bottle-25\_45;1666240;;calt0om;stability | 1666240 | calt0om | 16864 | 98.8 | 101.3 | 97.6 | calibration, time zero |
| gabapentin\_tablet-oralmix\_a\_bottle-25\_45;1678487;;calt0om;stability | 1678487 | calt0om | 16864 | 99.5 | 101.3 | 98.3 | calibration, time zero |
| gabapentin\_tablet-oralmix\_a\_bottle-25\_45;1670447;;calt0om;stability | 1670447 | calt0om | 16864 | 99.1 | 101.3 | 97.8 | calibration, time zero |
| gabapentin\_tablet-oralmix\_a\_bottle-25\_45;1668111;;calt0om;stability | 1668111 | calt0om | 16864 | 98.9 | 101.3 | 97.7 | calibration, time zero |
