# Supplementary material for: Stability of gabapentin in extemporaneously compounded oral suspensions
Source: PLoS One. 2017 Apr 17;12(4):e0175208. doi: 10.1371/journal.pone.0175208 (PMC5393583; doi:10.1371/journal.pone.0175208)
Supplement: S2 Appendix — Archive containing the HPLC stability results as browsable html pages. (ZIP) [file pone.0175208.s003.zip › gaba_s2_html_results/gabapentin/index.html?preparation=tablet-oralmix&lot=a&condition=bottle-25&time=60.html]

Stability Study Cruncher


### Preparation: tablet-oralmix, Lot: a, Condition: bottle-25, Time: 60

Assay (mg/mL): 96.7 ± 2.0 (n = 6);
Assay (%TZ): 95.5 ± 2.0 (n = 6).

| Input String | Area | Cal Id | Cal Slope | Assay | Assay TZ | Assay %TZ |  |
| --- | --- | --- | --- | --- | --- | --- | --- |
| gabapentin\_tablet-oralmix\_a\_bottle-25\_60;1625432;;calt0om;stability | 1625432 | calt0om | 16864 | 96.4 | 101.3 | 95.2 | calibration, time zero |
| gabapentin\_tablet-oralmix\_a\_bottle-25\_60;1625842;;calt0om;stability | 1625842 | calt0om | 16864 | 96.4 | 101.3 | 95.2 | calibration, time zero |
| gabapentin\_tablet-oralmix\_a\_bottle-25\_60;1615563;;calt0om;stability | 1615563 | calt0om | 16864 | 95.8 | 101.3 | 94.6 | calibration, time zero |
| gabapentin\_tablet-oralmix\_a\_bottle-25\_60;1698572;;calt0om;stability | 1698572 | calt0om | 16864 | 100.7 | 101.3 | 99.5 | calibration, time zero |
| gabapentin\_tablet-oralmix\_a\_bottle-25\_60;1614105;;calt0om;stability | 1614105 | calt0om | 16864 | 95.7 | 101.3 | 94.5 | calibration, time zero |
| gabapentin\_tablet-oralmix\_a\_bottle-25\_60;1609966;;calt0om;stability | 1609966 | calt0om | 16864 | 95.5 | 101.3 | 94.3 | calibration, time zero |
