# Supplementary material for: Stability of gabapentin in extemporaneously compounded oral suspensions
Source: PLoS One. 2017 Apr 17;12(4):e0175208. doi: 10.1371/journal.pone.0175208 (PMC5393583; doi:10.1371/journal.pone.0175208)
Supplement: S2 Appendix — Archive containing the HPLC stability results as browsable html pages. (ZIP) [file pone.0175208.s003.zip › gaba_s2_html_results/gabapentin/index.html?preparation=tablet-oralmix&lot=a&condition=bottle-25&time=75.html]

Stability Study Cruncher


### Preparation: tablet-oralmix, Lot: a, Condition: bottle-25, Time: 75

Assay (mg/mL): 96.4 ± 0.9 (n = 6);
Assay (%TZ): 95.2 ± 0.9 (n = 6).

| Input String | Area | Cal Id | Cal Slope | Assay | Assay TZ | Assay %TZ |  |
| --- | --- | --- | --- | --- | --- | --- | --- |
| gabapentin\_tablet-oralmix\_a\_bottle-25\_75;1634737;;calt0om;stability | 1634737 | calt0om | 16864 | 96.9 | 101.3 | 95.7 | calibration, time zero |
| gabapentin\_tablet-oralmix\_a\_bottle-25\_75;1636097;;calt0om;stability | 1636097 | calt0om | 16864 | 97.0 | 101.3 | 95.8 | calibration, time zero |
| gabapentin\_tablet-oralmix\_a\_bottle-25\_75;1637103;;calt0om;stability | 1637103 | calt0om | 16864 | 97.1 | 101.3 | 95.9 | calibration, time zero |
| gabapentin\_tablet-oralmix\_a\_bottle-25\_75;1637681;;calt0om;stability | 1637681 | calt0om | 16864 | 97.1 | 101.3 | 95.9 | calibration, time zero |
| gabapentin\_tablet-oralmix\_a\_bottle-25\_75;1605893;;calt0om;stability | 1605893 | calt0om | 16864 | 95.2 | 101.3 | 94.0 | calibration, time zero |
| gabapentin\_tablet-oralmix\_a\_bottle-25\_75;1606976;;calt0om;stability | 1606976 | calt0om | 16864 | 95.3 | 101.3 | 94.1 | calibration, time zero |
