# Supplementary material for: Stability of gabapentin in extemporaneously compounded oral suspensions
Source: PLoS One. 2017 Apr 17;12(4):e0175208. doi: 10.1371/journal.pone.0175208 (PMC5393583; doi:10.1371/journal.pone.0175208)
Supplement: S2 Appendix — Archive containing the HPLC stability results as browsable html pages. (ZIP) [file pone.0175208.s003.zip › gaba_s2_html_results/gabapentin/index.html?preparation=tablet-oralmix&lot=a&condition=bottle-25&time=90.html]

Stability Study Cruncher


### Preparation: tablet-oralmix, Lot: a, Condition: bottle-25, Time: 90

Assay (mg/mL): 94.3 ± 0.4 (n = 6);
Assay (%TZ): 93.1 ± 0.4 (n = 6).

| Input String | Area | Cal Id | Cal Slope | Assay | Assay TZ | Assay %TZ |  |
| --- | --- | --- | --- | --- | --- | --- | --- |
| gabapentin\_tablet-oralmix\_a\_bottle-25\_90;1599300;;calt0om;stability | 1599300 | calt0om | 16864 | 94.8 | 101.3 | 93.7 | calibration, time zero |
| gabapentin\_tablet-oralmix\_a\_bottle-25\_90;1597499;;calt0om;stability | 1597499 | calt0om | 16864 | 94.7 | 101.3 | 93.5 | calibration, time zero |
| gabapentin\_tablet-oralmix\_a\_bottle-25\_90;1586520;;calt0om;stability | 1586520 | calt0om | 16864 | 94.1 | 101.3 | 92.9 | calibration, time zero |
| gabapentin\_tablet-oralmix\_a\_bottle-25\_90;1582554;;calt0om;stability | 1582554 | calt0om | 16864 | 93.8 | 101.3 | 92.7 | calibration, time zero |
| gabapentin\_tablet-oralmix\_a\_bottle-25\_90;1584647;;calt0om;stability | 1584647 | calt0om | 16864 | 94.0 | 101.3 | 92.8 | calibration, time zero |
| gabapentin\_tablet-oralmix\_a\_bottle-25\_90;1587714;;calt0om;stability | 1587714 | calt0om | 16864 | 94.1 | 101.3 | 93.0 | calibration, time zero |
