# Supplementary material for: Stability of gabapentin in extemporaneously compounded oral suspensions
Source: PLoS One. 2017 Apr 17;12(4):e0175208. doi: 10.1371/journal.pone.0175208 (PMC5393583; doi:10.1371/journal.pone.0175208)
Supplement: S2 Appendix — Archive containing the HPLC stability results as browsable html pages. (ZIP) [file pone.0175208.s003.zip › gaba_s2_html_results/gabapentin/index.html?preparation=bulk-oralmixsf&lot=a.html]

Stability Study Cruncher


### Preparation: bulk-oralmixsf, Lot: a

Assay: 106.8 ± 0.9 mg/mL (n = 12).

| Input String | Area | Cal Id | Cal Slope | Assay |  |
| --- | --- | --- | --- | --- | --- |
| gabapentin\_bulk-oralmixsf\_a\_bottle-25;1684215;;calt0sf;time zero | 1684215 | calt0sf | 15817 | 106.5 | calibration |
| gabapentin\_bulk-oralmixsf\_a\_bottle-25;1682577;;calt0sf;time zero | 1682577 | calt0sf | 15817 | 106.4 | calibration |
| gabapentin\_bulk-oralmixsf\_a\_bottle-25;1672800;;calt0sf;time zero | 1672800 | calt0sf | 15817 | 105.8 | calibration |
| gabapentin\_bulk-oralmixsf\_a\_bottle-25;1674905;;calt0sf;time zero | 1674905 | calt0sf | 15817 | 105.9 | calibration |
| gabapentin\_bulk-oralmixsf\_a\_bottle-25;1710337;;calt0sf;time zero | 1710337 | calt0sf | 15817 | 108.1 | calibration |
| gabapentin\_bulk-oralmixsf\_a\_bottle-25;1709823;;calt0sf;time zero | 1709823 | calt0sf | 15817 | 108.1 | calibration |
| gabapentin\_bulk-oralmixsf\_a\_syringe-25;1689740;;calt0sf;time zero | 1689740 | calt0sf | 15817 | 106.8 | calibration |
| gabapentin\_bulk-oralmixsf\_a\_syringe-25;1688549;;calt0sf;time zero | 1688549 | calt0sf | 15817 | 106.8 | calibration |
| gabapentin\_bulk-oralmixsf\_a\_syringe-25;1678876;;calt0sf;time zero | 1678876 | calt0sf | 15817 | 106.1 | calibration |
| gabapentin\_bulk-oralmixsf\_a\_syringe-25;1679702;;calt0sf;time zero | 1679702 | calt0sf | 15817 | 106.2 | calibration |
| gabapentin\_bulk-oralmixsf\_a\_syringe-25;1702908;;calt0sf;time zero | 1702908 | calt0sf | 15817 | 107.7 | calibration |
| gabapentin\_bulk-oralmixsf\_a\_syringe-25;1705092;;calt0sf;time zero | 1705092 | calt0sf | 15817 | 107.8 | calibration |
