# Supplementary material for: Stability of gabapentin in extemporaneously compounded oral suspensions
Source: PLoS One. 2017 Apr 17;12(4):e0175208. doi: 10.1371/journal.pone.0175208 (PMC5393583; doi:10.1371/journal.pone.0175208)
Supplement: S2 Appendix — Archive containing the HPLC stability results as browsable html pages. (ZIP) [file pone.0175208.s003.zip › gaba_s2_html_results/gabapentin/index.html?preparation=tablet-oralmix&lot=a&condition=syringe-25&time=7.html]

Stability Study Cruncher


### Preparation: tablet-oralmix, Lot: a, Condition: syringe-25, Time: 7

Assay (mg/mL): 96.7 ± 2.6 (n = 6);
Assay (%TZ): 95.5 ± 2.6 (n = 6).

| Input String | Area | Cal Id | Cal Slope | Assay | Assay TZ | Assay %TZ |  |
| --- | --- | --- | --- | --- | --- | --- | --- |
| gabapentin\_tablet-oralmix\_a\_syringe-25\_7;1628714;;calt0om;stability | 1628714 | calt0om | 16864 | 96.6 | 101.3 | 95.4 | calibration, time zero |
| gabapentin\_tablet-oralmix\_a\_syringe-25\_7;1620279;;calt0om;stability | 1620279 | calt0om | 16864 | 96.1 | 101.3 | 94.9 | calibration, time zero |
| gabapentin\_tablet-oralmix\_a\_syringe-25\_7;1596951;;calt0om;stability | 1596951 | calt0om | 16864 | 94.7 | 101.3 | 93.5 | calibration, time zero |
| gabapentin\_tablet-oralmix\_a\_syringe-25\_7;1572979;;calt0om;stability | 1572979 | calt0om | 16864 | 93.3 | 101.3 | 92.1 | calibration, time zero |
| gabapentin\_tablet-oralmix\_a\_syringe-25\_7;1690315;;calt0om;stability | 1690315 | calt0om | 16864 | 100.2 | 101.3 | 99.0 | calibration, time zero |
| gabapentin\_tablet-oralmix\_a\_syringe-25\_7;1671048;;calt0om;stability | 1671048 | calt0om | 16864 | 99.1 | 101.3 | 97.9 | calibration, time zero |
