# Supplementary material for: Stability of gabapentin in extemporaneously compounded oral suspensions
Source: PLoS One. 2017 Apr 17;12(4):e0175208. doi: 10.1371/journal.pone.0175208 (PMC5393583; doi:10.1371/journal.pone.0175208)
Supplement: S2 Appendix — Archive containing the HPLC stability results as browsable html pages. (ZIP) [file pone.0175208.s003.zip › gaba_s2_html_results/gabapentin/index.html?preparation=tablet-oralmixsf&lot=a&condition=bottle-25&time=14.html]

Stability Study Cruncher


### Preparation: tablet-oralmixsf, Lot: a, Condition: bottle-25, Time: 14

Assay (mg/mL): 107.4 ± 2.8 (n = 6);
Assay (%TZ): 101.6 ± 2.7 (n = 6).

| Input String | Area | Cal Id | Cal Slope | Assay | Assay TZ | Assay %TZ |  |
| --- | --- | --- | --- | --- | --- | --- | --- |
| gabapentin\_tablet-oralmixsf\_a\_bottle-25\_14;1644840;;calt0sf;stability | 1644840 | calt0sf | 15817 | 104.0 | 105.7 | 98.4 | calibration, time zero |
| gabapentin\_tablet-oralmixsf\_a\_bottle-25\_14;1647790;;calt0sf;stability | 1647790 | calt0sf | 15817 | 104.2 | 105.7 | 98.6 | calibration, time zero |
| gabapentin\_tablet-oralmixsf\_a\_bottle-25\_14;1743886;;calt0sf;stability | 1743886 | calt0sf | 15817 | 110.3 | 105.7 | 104.3 | calibration, time zero |
| gabapentin\_tablet-oralmixsf\_a\_bottle-25\_14;1745745;;calt0sf;stability | 1745745 | calt0sf | 15817 | 110.4 | 105.7 | 104.4 | calibration, time zero |
| gabapentin\_tablet-oralmixsf\_a\_bottle-25\_14;1704609;;calt0sf;stability | 1704609 | calt0sf | 15817 | 107.8 | 105.7 | 102.0 | calibration, time zero |
| gabapentin\_tablet-oralmixsf\_a\_bottle-25\_14;1706400;;calt0sf;stability | 1706400 | calt0sf | 15817 | 107.9 | 105.7 | 102.1 | calibration, time zero |
