# Supplementary material for: Stability of gabapentin in extemporaneously compounded oral suspensions
Source: PLoS One. 2017 Apr 17;12(4):e0175208. doi: 10.1371/journal.pone.0175208 (PMC5393583; doi:10.1371/journal.pone.0175208)
Supplement: S2 Appendix — Archive containing the HPLC stability results as browsable html pages. (ZIP) [file pone.0175208.s003.zip › gaba_s2_html_results/gabapentin/index.html?preparation=tablet-oralmix&lot=a&condition=syringe-25&time=30.html]

Stability Study Cruncher


### Preparation: tablet-oralmix, Lot: a, Condition: syringe-25, Time: 30

Assay (mg/mL): 99.7 ± 0.8 (n = 6);
Assay (%TZ): 98.5 ± 0.8 (n = 6).

| Input String | Area | Cal Id | Cal Slope | Assay | Assay TZ | Assay %TZ |  |
| --- | --- | --- | --- | --- | --- | --- | --- |
| gabapentin\_tablet-oralmix\_a\_syringe-25\_30;1697042;;calt0om;stability | 1697042 | calt0om | 16864 | 100.6 | 101.3 | 99.4 | calibration, time zero |
| gabapentin\_tablet-oralmix\_a\_syringe-25\_30;1698920;;calt0om;stability | 1698920 | calt0om | 16864 | 100.7 | 101.3 | 99.5 | calibration, time zero |
| gabapentin\_tablet-oralmix\_a\_syringe-25\_30;1669863;;calt0om;stability | 1669863 | calt0om | 16864 | 99.0 | 101.3 | 97.8 | calibration, time zero |
| gabapentin\_tablet-oralmix\_a\_syringe-25\_30;1670889;;calt0om;stability | 1670889 | calt0om | 16864 | 99.1 | 101.3 | 97.8 | calibration, time zero |
| gabapentin\_tablet-oralmix\_a\_syringe-25\_30;1671042;;calt0om;stability | 1671042 | calt0om | 16864 | 99.1 | 101.3 | 97.9 | calibration, time zero |
| gabapentin\_tablet-oralmix\_a\_syringe-25\_30;1679942;;calt0om;stability | 1679942 | calt0om | 16864 | 99.6 | 101.3 | 98.4 | calibration, time zero |
