# Supplementary material for: Stability of gabapentin in extemporaneously compounded oral suspensions
Source: PLoS One. 2017 Apr 17;12(4):e0175208. doi: 10.1371/journal.pone.0175208 (PMC5393583; doi:10.1371/journal.pone.0175208)
Supplement: S2 Appendix — Archive containing the HPLC stability results as browsable html pages. (ZIP) [file pone.0175208.s003.zip › gaba_s2_html_results/gabapentin/index.html?preparation=tablet-oralmix&lot=a&condition=syringe-25&time=45.html]

Stability Study Cruncher


### Preparation: tablet-oralmix, Lot: a, Condition: syringe-25, Time: 45

Assay (mg/mL): 96.7 ± 2.1 (n = 6);
Assay (%TZ): 95.5 ± 2.0 (n = 6).

| Input String | Area | Cal Id | Cal Slope | Assay | Assay TZ | Assay %TZ |  |
| --- | --- | --- | --- | --- | --- | --- | --- |
| gabapentin\_tablet-oralmix\_a\_syringe-25\_45;1625483;;calt0om;stability | 1625483 | calt0om | 16864 | 96.4 | 101.3 | 95.2 | calibration, time zero |
| gabapentin\_tablet-oralmix\_a\_syringe-25\_45;1646607;;calt0om;stability | 1646607 | calt0om | 16864 | 97.6 | 101.3 | 96.4 | calibration, time zero |
| gabapentin\_tablet-oralmix\_a\_syringe-25\_45;1588549;;calt0om;stability | 1588549 | calt0om | 16864 | 94.2 | 101.3 | 93.0 | calibration, time zero |
| gabapentin\_tablet-oralmix\_a\_syringe-25\_45;1594593;;calt0om;stability | 1594593 | calt0om | 16864 | 94.6 | 101.3 | 93.4 | calibration, time zero |
| gabapentin\_tablet-oralmix\_a\_syringe-25\_45;1656229;;calt0om;stability | 1656229 | calt0om | 16864 | 98.2 | 101.3 | 97.0 | calibration, time zero |
| gabapentin\_tablet-oralmix\_a\_syringe-25\_45;1676234;;calt0om;stability | 1676234 | calt0om | 16864 | 99.4 | 101.3 | 98.2 | calibration, time zero |
