# Supplementary material for: Stability of gabapentin in extemporaneously compounded oral suspensions
Source: PLoS One. 2017 Apr 17;12(4):e0175208. doi: 10.1371/journal.pone.0175208 (PMC5393583; doi:10.1371/journal.pone.0175208)
Supplement: S2 Appendix — Archive containing the HPLC stability results as browsable html pages. (ZIP) [file pone.0175208.s003.zip › gaba_s2_html_results/gabapentin/index.html?preparation=tablet-oralmix&lot=a&condition=syringe-25&time=60.html]

Stability Study Cruncher


### Preparation: tablet-oralmix, Lot: a, Condition: syringe-25, Time: 60

Assay (mg/mL): 95.3 ± 1.1 (n = 6);
Assay (%TZ): 94.1 ± 1.1 (n = 6).

| Input String | Area | Cal Id | Cal Slope | Assay | Assay TZ | Assay %TZ |  |
| --- | --- | --- | --- | --- | --- | --- | --- |
| gabapentin\_tablet-oralmix\_a\_syringe-25\_60;1618794;;calt0om;stability | 1618794 | calt0om | 16864 | 96.0 | 101.3 | 94.8 | calibration, time zero |
| gabapentin\_tablet-oralmix\_a\_syringe-25\_60;1617670;;calt0om;stability | 1617670 | calt0om | 16864 | 95.9 | 101.3 | 94.7 | calibration, time zero |
| gabapentin\_tablet-oralmix\_a\_syringe-25\_60;1586575;;calt0om;stability | 1586575 | calt0om | 16864 | 94.1 | 101.3 | 92.9 | calibration, time zero |
| gabapentin\_tablet-oralmix\_a\_syringe-25\_60;1581019;;calt0om;stability | 1581019 | calt0om | 16864 | 93.8 | 101.3 | 92.6 | calibration, time zero |
| gabapentin\_tablet-oralmix\_a\_syringe-25\_60;1622052;;calt0om;stability | 1622052 | calt0om | 16864 | 96.2 | 101.3 | 95.0 | calibration, time zero |
| gabapentin\_tablet-oralmix\_a\_syringe-25\_60;1620153;;calt0om;stability | 1620153 | calt0om | 16864 | 96.1 | 101.3 | 94.9 | calibration, time zero |
