# Supplementary material for: Stability of gabapentin in extemporaneously compounded oral suspensions
Source: PLoS One. 2017 Apr 17;12(4):e0175208. doi: 10.1371/journal.pone.0175208 (PMC5393583; doi:10.1371/journal.pone.0175208)
Supplement: S2 Appendix — Archive containing the HPLC stability results as browsable html pages. (ZIP) [file pone.0175208.s003.zip › gaba_s2_html_results/gabapentin/index.html?preparation=tablet-oralmixsf&lot=a.html]

Stability Study Cruncher


### Preparation: tablet-oralmixsf, Lot: a

Assay: 105.7 ± 0.8 mg/mL (n = 12).

| Input String | Area | Cal Id | Cal Slope | Assay |  |
| --- | --- | --- | --- | --- | --- |
| gabapentin\_tablet-oralmixsf\_a\_bottle-25;1671759;;calt0sf;time zero | 1671759 | calt0sf | 15817 | 105.7 | calibration |
| gabapentin\_tablet-oralmixsf\_a\_bottle-25;1670184;;calt0sf;time zero | 1670184 | calt0sf | 15817 | 105.6 | calibration |
| gabapentin\_tablet-oralmixsf\_a\_bottle-25;1679520;;calt0sf;time zero | 1679520 | calt0sf | 15817 | 106.2 | calibration |
| gabapentin\_tablet-oralmixsf\_a\_bottle-25;1677695;;calt0sf;time zero | 1677695 | calt0sf | 15817 | 106.1 | calibration |
| gabapentin\_tablet-oralmixsf\_a\_bottle-25;1648536;;calt0sf;time zero | 1648536 | calt0sf | 15817 | 104.2 | calibration |
| gabapentin\_tablet-oralmixsf\_a\_bottle-25;1644193;;calt0sf;time zero | 1644193 | calt0sf | 15817 | 103.9 | calibration |
| gabapentin\_tablet-oralmixsf\_a\_syringe-25;1692708;;calt0sf;time zero | 1692708 | calt0sf | 15817 | 107.0 | calibration |
| gabapentin\_tablet-oralmixsf\_a\_syringe-25;1681687;;calt0sf;time zero | 1681687 | calt0sf | 15817 | 106.3 | calibration |
| gabapentin\_tablet-oralmixsf\_a\_syringe-25;1671906;;calt0sf;time zero | 1671906 | calt0sf | 15817 | 105.7 | calibration |
| gabapentin\_tablet-oralmixsf\_a\_syringe-25;1672144;;calt0sf;time zero | 1672144 | calt0sf | 15817 | 105.7 | calibration |
| gabapentin\_tablet-oralmixsf\_a\_syringe-25;1673710;;calt0sf;time zero | 1673710 | calt0sf | 15817 | 105.8 | calibration |
| gabapentin\_tablet-oralmixsf\_a\_syringe-25;1676296;;calt0sf;time zero | 1676296 | calt0sf | 15817 | 106.0 | calibration |
