# Supplementary material for: Stability of gabapentin in extemporaneously compounded oral suspensions
Source: PLoS One. 2017 Apr 17;12(4):e0175208. doi: 10.1371/journal.pone.0175208 (PMC5393583; doi:10.1371/journal.pone.0175208)
Supplement: S2 Appendix — Archive containing the HPLC stability results as browsable html pages. (ZIP) [file pone.0175208.s003.zip › gaba_s2_html_results/gabapentin/index.html?preparation=bulk-oralmixsf&lot=a&condition=bottle-25&time=7.html]

Stability Study Cruncher


### Preparation: bulk-oralmixsf, Lot: a, Condition: bottle-25, Time: 7

Assay (mg/mL): 107.7 ± 0.8 (n = 6);
Assay (%TZ): 100.8 ± 0.8 (n = 6).

| Input String | Area | Cal Id | Cal Slope | Assay | Assay TZ | Assay %TZ |  |
| --- | --- | --- | --- | --- | --- | --- | --- |
| gabapentin\_bulk-oralmixsf\_a\_bottle-25\_7;1720270;;calt0sf;stability | 1720270 | calt0sf | 15817 | 108.8 | 106.8 | 101.8 | calibration, time zero |
| gabapentin\_bulk-oralmixsf\_a\_bottle-25\_7;1720611;;calt0sf;stability | 1720611 | calt0sf | 15817 | 108.8 | 106.8 | 101.8 | calibration, time zero |
| gabapentin\_bulk-oralmixsf\_a\_bottle-25\_7;1701004;;calt0sf;stability | 1701004 | calt0sf | 15817 | 107.5 | 106.8 | 100.7 | calibration, time zero |
| gabapentin\_bulk-oralmixsf\_a\_bottle-25\_7;1700017;;calt0sf;stability | 1700017 | calt0sf | 15817 | 107.5 | 106.8 | 100.6 | calibration, time zero |
| gabapentin\_bulk-oralmixsf\_a\_bottle-25\_7;1692694;;calt0sf;stability | 1692694 | calt0sf | 15817 | 107.0 | 106.8 | 100.2 | calibration, time zero |
| gabapentin\_bulk-oralmixsf\_a\_bottle-25\_7;1690230;;calt0sf;stability | 1690230 | calt0sf | 15817 | 106.9 | 106.8 | 100.0 | calibration, time zero |
