# Supplementary material for: Stability of gabapentin in extemporaneously compounded oral suspensions
Source: PLoS One. 2017 Apr 17;12(4):e0175208. doi: 10.1371/journal.pone.0175208 (PMC5393583; doi:10.1371/journal.pone.0175208)
Supplement: S2 Appendix — Archive containing the HPLC stability results as browsable html pages. (ZIP) [file pone.0175208.s003.zip › gaba_s2_html_results/gabapentin/index.html?preparation=tablet-oralmix&lot=a&condition=syringe-25&time=90.html]

Stability Study Cruncher


### Preparation: tablet-oralmix, Lot: a, Condition: syringe-25, Time: 90

Assay (mg/mL): 96.4 ± 1.8 (n = 6);
Assay (%TZ): 95.2 ± 1.8 (n = 6).

| Input String | Area | Cal Id | Cal Slope | Assay | Assay TZ | Assay %TZ |  |
| --- | --- | --- | --- | --- | --- | --- | --- |
| gabapentin\_tablet-oralmix\_a\_syringe-25\_90;1625840;;calt0om;stability | 1625840 | calt0om | 16864 | 96.4 | 101.3 | 95.2 | calibration, time zero |
| gabapentin\_tablet-oralmix\_a\_syringe-25\_90;1625109;;calt0om;stability | 1625109 | calt0om | 16864 | 96.4 | 101.3 | 95.2 | calibration, time zero |
| gabapentin\_tablet-oralmix\_a\_syringe-25\_90;1585588;;calt0om;stability | 1585588 | calt0om | 16864 | 94.0 | 101.3 | 92.8 | calibration, time zero |
| gabapentin\_tablet-oralmix\_a\_syringe-25\_90;1600632;;calt0om;stability | 1600632 | calt0om | 16864 | 94.9 | 101.3 | 93.7 | calibration, time zero |
| gabapentin\_tablet-oralmix\_a\_syringe-25\_90;1658220;;calt0om;stability | 1658220 | calt0om | 16864 | 98.3 | 101.3 | 97.1 | calibration, time zero |
| gabapentin\_tablet-oralmix\_a\_syringe-25\_90;1662769;;calt0om;stability | 1662769 | calt0om | 16864 | 98.6 | 101.3 | 97.4 | calibration, time zero |
