# Supplementary material for: Stability of gabapentin in extemporaneously compounded oral suspensions
Source: PLoS One. 2017 Apr 17;12(4):e0175208. doi: 10.1371/journal.pone.0175208 (PMC5393583; doi:10.1371/journal.pone.0175208)
Supplement: S2 Appendix — Archive containing the HPLC stability results as browsable html pages. (ZIP) [file pone.0175208.s003.zip › gaba_s2_html_results/gabapentin/index.html?preparation=bulk-oralmixsf&lot=a&condition=bottle-25&time=14.html]

Stability Study Cruncher


### Preparation: bulk-oralmixsf, Lot: a, Condition: bottle-25, Time: 14

Assay (mg/mL): 110.9 ± 2.6 (n = 6);
Assay (%TZ): 103.8 ± 2.4 (n = 6).

| Input String | Area | Cal Id | Cal Slope | Assay | Assay TZ | Assay %TZ |  |
| --- | --- | --- | --- | --- | --- | --- | --- |
| gabapentin\_bulk-oralmixsf\_a\_bottle-25\_14;1802204;;calt0sf;stability | 1802204 | calt0sf | 15817 | 113.9 | 106.8 | 106.6 | calibration, time zero |
| gabapentin\_bulk-oralmixsf\_a\_bottle-25\_14;1805442;;calt0sf;stability | 1805442 | calt0sf | 15817 | 114.1 | 106.8 | 106.8 | calibration, time zero |
| gabapentin\_bulk-oralmixsf\_a\_bottle-25\_14;1712801;;calt0sf;stability | 1712801 | calt0sf | 15817 | 108.3 | 106.8 | 101.4 | calibration, time zero |
| gabapentin\_bulk-oralmixsf\_a\_bottle-25\_14;1713354;;calt0sf;stability | 1713354 | calt0sf | 15817 | 108.3 | 106.8 | 101.4 | calibration, time zero |
| gabapentin\_bulk-oralmixsf\_a\_bottle-25\_14;1744510;;calt0sf;stability | 1744510 | calt0sf | 15817 | 110.3 | 106.8 | 103.2 | calibration, time zero |
| gabapentin\_bulk-oralmixsf\_a\_bottle-25\_14;1747559;;calt0sf;stability | 1747559 | calt0sf | 15817 | 110.5 | 106.8 | 103.4 | calibration, time zero |
