# Supplementary material for: Stability of gabapentin in extemporaneously compounded oral suspensions
Source: PLoS One. 2017 Apr 17;12(4):e0175208. doi: 10.1371/journal.pone.0175208 (PMC5393583; doi:10.1371/journal.pone.0175208)
Supplement: S2 Appendix — Archive containing the HPLC stability results as browsable html pages. (ZIP) [file pone.0175208.s003.zip › gaba_s2_html_results/gabapentin/index.html?preparation=bulk-oralmixsf&lot=a&condition=bottle-25&time=30.html]

Stability Study Cruncher


### Preparation: bulk-oralmixsf, Lot: a, Condition: bottle-25, Time: 30

Assay (mg/mL): 111.5 ± 1.4 (n = 6);
Assay (%TZ): 104.3 ± 1.3 (n = 6).

| Input String | Area | Cal Id | Cal Slope | Assay | Assay TZ | Assay %TZ |  |
| --- | --- | --- | --- | --- | --- | --- | --- |
| gabapentin\_bulk-oralmixsf\_a\_bottle-25\_30;1735878;;calt0sf;stability | 1735878 | calt0sf | 15817 | 109.7 | 106.8 | 102.7 | calibration, time zero |
| gabapentin\_bulk-oralmixsf\_a\_bottle-25\_30;1736222;;calt0sf;stability | 1736222 | calt0sf | 15817 | 109.8 | 106.8 | 102.7 | calibration, time zero |
| gabapentin\_bulk-oralmixsf\_a\_bottle-25\_30;1781229;;calt0sf;stability | 1781229 | calt0sf | 15817 | 112.6 | 106.8 | 105.4 | calibration, time zero |
| gabapentin\_bulk-oralmixsf\_a\_bottle-25\_30;1782558;;calt0sf;stability | 1782558 | calt0sf | 15817 | 112.7 | 106.8 | 105.5 | calibration, time zero |
| gabapentin\_bulk-oralmixsf\_a\_bottle-25\_30;1770115;;calt0sf;stability | 1770115 | calt0sf | 15817 | 111.9 | 106.8 | 104.7 | calibration, time zero |
| gabapentin\_bulk-oralmixsf\_a\_bottle-25\_30;1771068;;calt0sf;stability | 1771068 | calt0sf | 15817 | 112.0 | 106.8 | 104.8 | calibration, time zero |
