# Supplementary material for: Stability of gabapentin in extemporaneously compounded oral suspensions
Source: PLoS One. 2017 Apr 17;12(4):e0175208. doi: 10.1371/journal.pone.0175208 (PMC5393583; doi:10.1371/journal.pone.0175208)
Supplement: S2 Appendix — Archive containing the HPLC stability results as browsable html pages. (ZIP) [file pone.0175208.s003.zip › gaba_s2_html_results/gabapentin/index.html?preparation=bulk-oralmixsf&lot=a&condition=bottle-25&time=45.html]

Stability Study Cruncher


### Preparation: bulk-oralmixsf, Lot: a, Condition: bottle-25, Time: 45

Assay (mg/mL): 104.7 ± 1.3 (n = 6);
Assay (%TZ): 98.0 ± 1.2 (n = 6).

| Input String | Area | Cal Id | Cal Slope | Assay | Assay TZ | Assay %TZ |  |
| --- | --- | --- | --- | --- | --- | --- | --- |
| gabapentin\_bulk-oralmixsf\_a\_bottle-25\_45;1639729;;calt45sf;stability | 1639729 | calt45sf | 15852 | 103.4 | 106.8 | 96.8 | calibration, time zero |
| gabapentin\_bulk-oralmixsf\_a\_bottle-25\_45;1641026;;calt45sf;stability | 1641026 | calt45sf | 15852 | 103.5 | 106.8 | 96.9 | calibration, time zero |
| gabapentin\_bulk-oralmixsf\_a\_bottle-25\_45;1653544;;calt45sf;stability | 1653544 | calt45sf | 15852 | 104.3 | 106.8 | 97.6 | calibration, time zero |
| gabapentin\_bulk-oralmixsf\_a\_bottle-25\_45;1653555;;calt45sf;stability | 1653555 | calt45sf | 15852 | 104.3 | 106.8 | 97.6 | calibration, time zero |
| gabapentin\_bulk-oralmixsf\_a\_bottle-25\_45;1682889;;calt45sf;stability | 1682889 | calt45sf | 15852 | 106.2 | 106.8 | 99.4 | calibration, time zero |
| gabapentin\_bulk-oralmixsf\_a\_bottle-25\_45;1684602;;calt45sf;stability | 1684602 | calt45sf | 15852 | 106.3 | 106.8 | 99.5 | calibration, time zero |
