# Supplementary material for: Stability of gabapentin in extemporaneously compounded oral suspensions
Source: PLoS One. 2017 Apr 17;12(4):e0175208. doi: 10.1371/journal.pone.0175208 (PMC5393583; doi:10.1371/journal.pone.0175208)
Supplement: S2 Appendix — Archive containing the HPLC stability results as browsable html pages. (ZIP) [file pone.0175208.s003.zip › gaba_s2_html_results/gabapentin/index.html?preparation=bulk-oralmixsf&lot=a&condition=bottle-25&time=60.html]

Stability Study Cruncher


### Preparation: bulk-oralmixsf, Lot: a, Condition: bottle-25, Time: 60

Assay (mg/mL): 108.4 ± 2.7 (n = 6);
Assay (%TZ): 101.4 ± 2.6 (n = 6).

| Input String | Area | Cal Id | Cal Slope | Assay | Assay TZ | Assay %TZ |  |
| --- | --- | --- | --- | --- | --- | --- | --- |
| gabapentin\_bulk-oralmixsf\_a\_bottle-25\_60;1662466;;calt45sf;stability | 1662466 | calt45sf | 15852 | 104.9 | 106.8 | 98.2 | calibration, time zero |
| gabapentin\_bulk-oralmixsf\_a\_bottle-25\_60;1663998;;calt45sf;stability | 1663998 | calt45sf | 15852 | 105.0 | 106.8 | 98.2 | calibration, time zero |
| gabapentin\_bulk-oralmixsf\_a\_bottle-25\_60;1753402;;calt45sf;stability | 1753402 | calt45sf | 15852 | 110.6 | 106.8 | 103.5 | calibration, time zero |
| gabapentin\_bulk-oralmixsf\_a\_bottle-25\_60;1757257;;calt45sf;stability | 1757257 | calt45sf | 15852 | 110.9 | 106.8 | 103.8 | calibration, time zero |
| gabapentin\_bulk-oralmixsf\_a\_bottle-25\_60;1733428;;calt45sf;stability | 1733428 | calt45sf | 15852 | 109.3 | 106.8 | 102.3 | calibration, time zero |
| gabapentin\_bulk-oralmixsf\_a\_bottle-25\_60;1735643;;calt45sf;stability | 1735643 | calt45sf | 15852 | 109.5 | 106.8 | 102.5 | calibration, time zero |
