# Supplementary material for: Stability of gabapentin in extemporaneously compounded oral suspensions
Source: PLoS One. 2017 Apr 17;12(4):e0175208. doi: 10.1371/journal.pone.0175208 (PMC5393583; doi:10.1371/journal.pone.0175208)
Supplement: S2 Appendix — Archive containing the HPLC stability results as browsable html pages. (ZIP) [file pone.0175208.s003.zip › gaba_s2_html_results/gabapentin/index.html?preparation=bulk-oralmixsf&lot=a&condition=bottle-25&time=75.html]

Stability Study Cruncher


### Preparation: bulk-oralmixsf, Lot: a, Condition: bottle-25, Time: 75

Assay (mg/mL): 113.0 ± 4.0 (n = 6);
Assay (%TZ): 105.7 ± 3.8 (n = 6).

| Input String | Area | Cal Id | Cal Slope | Assay | Assay TZ | Assay %TZ |  |
| --- | --- | --- | --- | --- | --- | --- | --- |
| gabapentin\_bulk-oralmixsf\_a\_bottle-25\_75;1870974;;calt45sf;stability | 1870974 | calt45sf | 15852 | 118.0 | 106.8 | 110.5 | calibration, time zero |
| gabapentin\_bulk-oralmixsf\_a\_bottle-25\_75;1874731;;calt45sf;stability | 1874731 | calt45sf | 15852 | 118.3 | 106.8 | 110.7 | calibration, time zero |
| gabapentin\_bulk-oralmixsf\_a\_bottle-25\_75;1751393;;calt45sf;stability | 1751393 | calt45sf | 15852 | 110.5 | 106.8 | 103.4 | calibration, time zero |
| gabapentin\_bulk-oralmixsf\_a\_bottle-25\_75;1747241;;calt45sf;stability | 1747241 | calt45sf | 15852 | 110.2 | 106.8 | 103.2 | calibration, time zero |
| gabapentin\_bulk-oralmixsf\_a\_bottle-25\_75;1744000;;calt45sf;stability | 1744000 | calt45sf | 15852 | 110.0 | 106.8 | 103.0 | calibration, time zero |
| gabapentin\_bulk-oralmixsf\_a\_bottle-25\_75;1755463;;calt45sf;stability | 1755463 | calt45sf | 15852 | 110.7 | 106.8 | 103.6 | calibration, time zero |
