# Supplementary material for: Stability of gabapentin in extemporaneously compounded oral suspensions
Source: PLoS One. 2017 Apr 17;12(4):e0175208. doi: 10.1371/journal.pone.0175208 (PMC5393583; doi:10.1371/journal.pone.0175208)
Supplement: S2 Appendix — Archive containing the HPLC stability results as browsable html pages. (ZIP) [file pone.0175208.s003.zip › gaba_s2_html_results/gabapentin/index.html?preparation=bulk-oralmixsf&lot=a&condition=bottle-25&time=90.html]

Stability Study Cruncher


### Preparation: bulk-oralmixsf, Lot: a, Condition: bottle-25, Time: 90

Assay (mg/mL): 110.7 ± 0.5 (n = 6);
Assay (%TZ): 103.6 ± 0.5 (n = 6).

| Input String | Area | Cal Id | Cal Slope | Assay | Assay TZ | Assay %TZ |  |
| --- | --- | --- | --- | --- | --- | --- | --- |
| gabapentin\_bulk-oralmixsf\_a\_bottle-25\_90;1758446;;calt45sf;stability | 1758446 | calt45sf | 15852 | 110.9 | 106.8 | 103.8 | calibration, time zero |
| gabapentin\_bulk-oralmixsf\_a\_bottle-25\_90;1756403;;calt45sf;stability | 1756403 | calt45sf | 15852 | 110.8 | 106.8 | 103.7 | calibration, time zero |
| gabapentin\_bulk-oralmixsf\_a\_bottle-25\_90;1747064;;calt45sf;stability | 1747064 | calt45sf | 15852 | 110.2 | 106.8 | 103.1 | calibration, time zero |
| gabapentin\_bulk-oralmixsf\_a\_bottle-25\_90;1743397;;calt45sf;stability | 1743397 | calt45sf | 15852 | 110.0 | 106.8 | 102.9 | calibration, time zero |
| gabapentin\_bulk-oralmixsf\_a\_bottle-25\_90;1763381;;calt45sf;stability | 1763381 | calt45sf | 15852 | 111.2 | 106.8 | 104.1 | calibration, time zero |
| gabapentin\_bulk-oralmixsf\_a\_bottle-25\_90;1759881;;calt45sf;stability | 1759881 | calt45sf | 15852 | 111.0 | 106.8 | 103.9 | calibration, time zero |
