# Supplementary material for: Stability of gabapentin in extemporaneously compounded oral suspensions
Source: PLoS One. 2017 Apr 17;12(4):e0175208. doi: 10.1371/journal.pone.0175208 (PMC5393583; doi:10.1371/journal.pone.0175208)
Supplement: S2 Appendix — Archive containing the HPLC stability results as browsable html pages. (ZIP) [file pone.0175208.s003.zip › gaba_s2_html_results/gabapentin/index.html?preparation=bulk-oralmixsf&lot=a&condition=syringe-25&time=7.html]

Stability Study Cruncher


### Preparation: bulk-oralmixsf, Lot: a, Condition: syringe-25, Time: 7

Assay (mg/mL): 108.3 ± 1.5 (n = 6);
Assay (%TZ): 101.4 ± 1.4 (n = 6).

| Input String | Area | Cal Id | Cal Slope | Assay | Assay TZ | Assay %TZ |  |
| --- | --- | --- | --- | --- | --- | --- | --- |
| gabapentin\_bulk-oralmixsf\_a\_syringe-25\_7;1723109;;calt0sf;stability | 1723109 | calt0sf | 15817 | 108.9 | 106.8 | 102.0 | calibration, time zero |
| gabapentin\_bulk-oralmixsf\_a\_syringe-25\_7;1715704;;calt0sf;stability | 1715704 | calt0sf | 15817 | 108.5 | 106.8 | 101.5 | calibration, time zero |
| gabapentin\_bulk-oralmixsf\_a\_syringe-25\_7;1685192;;calt0sf;stability | 1685192 | calt0sf | 15817 | 106.5 | 106.8 | 99.7 | calibration, time zero |
| gabapentin\_bulk-oralmixsf\_a\_syringe-25\_7;1685085;;calt0sf;stability | 1685085 | calt0sf | 15817 | 106.5 | 106.8 | 99.7 | calibration, time zero |
| gabapentin\_bulk-oralmixsf\_a\_syringe-25\_7;1737396;;calt0sf;stability | 1737396 | calt0sf | 15817 | 109.8 | 106.8 | 102.8 | calibration, time zero |
| gabapentin\_bulk-oralmixsf\_a\_syringe-25\_7;1733346;;calt0sf;stability | 1733346 | calt0sf | 15817 | 109.6 | 106.8 | 102.6 | calibration, time zero |
