# Supplementary material for: Stability of gabapentin in extemporaneously compounded oral suspensions
Source: PLoS One. 2017 Apr 17;12(4):e0175208. doi: 10.1371/journal.pone.0175208 (PMC5393583; doi:10.1371/journal.pone.0175208)
Supplement: S2 Appendix — Archive containing the HPLC stability results as browsable html pages. (ZIP) [file pone.0175208.s003.zip › gaba_s2_html_results/gabapentin/index.html?calibrationId=calt0om.html]

Stability Study Cruncher


### Calibration Id: calt0om

Slope: 16864 1/mg/mL (r2 = 0.99999, n = 18).

|  |  |  |  |  |  |  |  |  |  |  |  |  |  |  |  |  |  |  |  |  |  |  |  |  |  |  |  |  |  |  |  |  |  |  |  |  |  |  |  |  |  |  |  |  |  |  |  |  |  |  |  |  |  |  |  |  |
| --- | --- | --- | --- | --- | --- | --- | --- | --- | --- | --- | --- | --- | --- | --- | --- | --- | --- | --- | --- | --- | --- | --- | --- | --- | --- | --- | --- | --- | --- | --- | --- | --- | --- | --- | --- | --- | --- | --- | --- | --- | --- | --- | --- | --- | --- | --- | --- | --- | --- | --- | --- | --- | --- | --- | --- | --- |
| Input String | Conc | Area |||  |  |  |  |  |  |  |  |  |  |  |  |  |  |  |  |  |  |  |  |  |  |  |  |  |  |  |  |  |  |  |  |  |  |  |  |  |  |  |  |  |  |  |  |  |  |  |  |  |  |  |  |  |  |
| --- | --- | --- | --- | --- | --- | --- | --- | --- | --- | --- | --- | --- | --- | --- | --- | --- | --- | --- | --- | --- | --- | --- | --- | --- | --- | --- | --- | --- | --- | --- | --- | --- | --- | --- | --- | --- | --- | --- | --- | --- | --- | --- | --- | --- | --- | --- | --- | --- | --- | --- | --- | --- | --- |
| gabapentin\_STD000;0;0;calt0om;calibration | 0.0 | 0 || gabapentin\_STD0.5;317156;18.528;calt0om;calibration | 18.5 | 317156 || gabapentin\_STD1.0;634427;37.056;calt0om;calibration | 37.1 | 634427 || gabapentin\_STD2.5;1568514;92.64;calt0om;calibration | 92.6 | 1568514 || gabapentin\_STD3.75;2354164;138.96;calt0om;calibration | 139.0 | 2354164 || gabapentin\_STD5.0;3121733;185.28;calt0om;calibration | 185.3 | 3121733 || gabapentin\_STD000;0;0;calt0om;calibration | 0.0 | 0 || gabapentin\_STD0.5;312695;18.528;calt0om;calibration | 18.5 | 312695 || gabapentin\_STD1.0;632901;37.056;calt0om;calibration | 37.1 | 632901 || gabapentin\_STD2.5;1565020;92.64;calt0om;calibration | 92.6 | 1565020 || gabapentin\_STD3.75;2349624;138.96;calt0om;calibration | 139.0 | 2349624 || gabapentin\_STD5.0;3125905;185.28;calt0om;calibration | 185.3 | 3125905 || gabapentin\_STD000;0;0;calt0om;calibration | 0.0 | 0 || gabapentin\_STD0.5;313400;18.528;calt0om;calibration | 18.5 | 313400 || gabapentin\_STD1.0;631304;37.056;calt0om;calibration | 37.1 | 631304 || gabapentin\_STD2.5;1557200;92.64;calt0om;calibration | 92.6 | 1557200 || gabapentin\_STD3.75;2335585;138.96;calt0om;calibration | 139.0 | 2335585 || gabapentin\_STD5.0;3111978;185.28;calt0om;calibration | 185.3 | 3111978 |
