# Supplementary material for: Stability of gabapentin in extemporaneously compounded oral suspensions
Source: PLoS One. 2017 Apr 17;12(4):e0175208. doi: 10.1371/journal.pone.0175208 (PMC5393583; doi:10.1371/journal.pone.0175208)
Supplement: S2 Appendix — Archive containing the HPLC stability results as browsable html pages. (ZIP) [file pone.0175208.s003.zip › gaba_s2_html_results/gabapentin/index.html?preparation=bulk-oralmixsf&lot=a&condition=syringe-25&time=14.html]

Stability Study Cruncher


### Preparation: bulk-oralmixsf, Lot: a, Condition: syringe-25, Time: 14

Assay (mg/mL): 109.6 ± 4.2 (n = 6);
Assay (%TZ): 102.5 ± 3.9 (n = 6).

| Input String | Area | Cal Id | Cal Slope | Assay | Assay TZ | Assay %TZ |  |
| --- | --- | --- | --- | --- | --- | --- | --- |
| gabapentin\_bulk-oralmixsf\_a\_syringe-25\_14;1799469;;calt0sf;stability | 1799469 | calt0sf | 15817 | 113.8 | 106.8 | 106.5 | calibration, time zero |
| gabapentin\_bulk-oralmixsf\_a\_syringe-25\_14;1803475;;calt0sf;stability | 1803475 | calt0sf | 15817 | 114.0 | 106.8 | 106.7 | calibration, time zero |
| gabapentin\_bulk-oralmixsf\_a\_syringe-25\_14;1738998;;calt0sf;stability | 1738998 | calt0sf | 15817 | 109.9 | 106.8 | 102.9 | calibration, time zero |
| gabapentin\_bulk-oralmixsf\_a\_syringe-25\_14;1744476;;calt0sf;stability | 1744476 | calt0sf | 15817 | 110.3 | 106.8 | 103.2 | calibration, time zero |
| gabapentin\_bulk-oralmixsf\_a\_syringe-25\_14;1653828;;calt0sf;stability | 1653828 | calt0sf | 15817 | 104.6 | 106.8 | 97.9 | calibration, time zero |
| gabapentin\_bulk-oralmixsf\_a\_syringe-25\_14;1656760;;calt0sf;stability | 1656760 | calt0sf | 15817 | 104.7 | 106.8 | 98.0 | calibration, time zero |
