# Supplementary material for: Stability of gabapentin in extemporaneously compounded oral suspensions
Source: PLoS One. 2017 Apr 17;12(4):e0175208. doi: 10.1371/journal.pone.0175208 (PMC5393583; doi:10.1371/journal.pone.0175208)
Supplement: S2 Appendix — Archive containing the HPLC stability results as browsable html pages. (ZIP) [file pone.0175208.s003.zip › gaba_s2_html_results/gabapentin/index.html?preparation=bulk-oralmixsf&lot=a&condition=syringe-25&time=30.html]

Stability Study Cruncher


### Preparation: bulk-oralmixsf, Lot: a, Condition: syringe-25, Time: 30

Assay (mg/mL): 110.6 ± 1.1 (n = 6);
Assay (%TZ): 103.5 ± 1.0 (n = 6).

| Input String | Area | Cal Id | Cal Slope | Assay | Assay TZ | Assay %TZ |  |
| --- | --- | --- | --- | --- | --- | --- | --- |
| gabapentin\_bulk-oralmixsf\_a\_syringe-25\_30;1755641;;calt0sf;stability | 1755641 | calt0sf | 15817 | 111.0 | 106.8 | 103.9 | calibration, time zero |
| gabapentin\_bulk-oralmixsf\_a\_syringe-25\_30;1758174;;calt0sf;stability | 1758174 | calt0sf | 15817 | 111.2 | 106.8 | 104.0 | calibration, time zero |
| gabapentin\_bulk-oralmixsf\_a\_syringe-25\_30;1762826;;calt0sf;stability | 1762826 | calt0sf | 15817 | 111.5 | 106.8 | 104.3 | calibration, time zero |
| gabapentin\_bulk-oralmixsf\_a\_syringe-25\_30;1764836;;calt0sf;stability | 1764836 | calt0sf | 15817 | 111.6 | 106.8 | 104.4 | calibration, time zero |
| gabapentin\_bulk-oralmixsf\_a\_syringe-25\_30;1728323;;calt0sf;stability | 1728323 | calt0sf | 15817 | 109.3 | 106.8 | 102.3 | calibration, time zero |
| gabapentin\_bulk-oralmixsf\_a\_syringe-25\_30;1728410;;calt0sf;stability | 1728410 | calt0sf | 15817 | 109.3 | 106.8 | 102.3 | calibration, time zero |
