# Supplementary material for: Stability of gabapentin in extemporaneously compounded oral suspensions
Source: PLoS One. 2017 Apr 17;12(4):e0175208. doi: 10.1371/journal.pone.0175208 (PMC5393583; doi:10.1371/journal.pone.0175208)
Supplement: S2 Appendix — Archive containing the HPLC stability results as browsable html pages. (ZIP) [file pone.0175208.s003.zip › gaba_s2_html_results/gabapentin/index.html?preparation=bulk-oralmixsf&lot=a&condition=syringe-25&time=45.html]

Stability Study Cruncher


### Preparation: bulk-oralmixsf, Lot: a, Condition: syringe-25, Time: 45

Assay (mg/mL): 106.9 ± 1.2 (n = 6);
Assay (%TZ): 100.0 ± 1.2 (n = 6).

| Input String | Area | Cal Id | Cal Slope | Assay | Assay TZ | Assay %TZ |  |
| --- | --- | --- | --- | --- | --- | --- | --- |
| gabapentin\_bulk-oralmixsf\_a\_syringe-25\_45;1674262;;calt45sf;stability | 1674262 | calt45sf | 15852 | 105.6 | 106.8 | 98.9 | calibration, time zero |
| gabapentin\_bulk-oralmixsf\_a\_syringe-25\_45;1677449;;calt45sf;stability | 1677449 | calt45sf | 15852 | 105.8 | 106.8 | 99.0 | calibration, time zero |
| gabapentin\_bulk-oralmixsf\_a\_syringe-25\_45;1687338;;calt45sf;stability | 1687338 | calt45sf | 15852 | 106.4 | 106.8 | 99.6 | calibration, time zero |
| gabapentin\_bulk-oralmixsf\_a\_syringe-25\_45;1688523;;calt45sf;stability | 1688523 | calt45sf | 15852 | 106.5 | 106.8 | 99.7 | calibration, time zero |
| gabapentin\_bulk-oralmixsf\_a\_syringe-25\_45;1713829;;calt45sf;stability | 1713829 | calt45sf | 15852 | 108.1 | 106.8 | 101.2 | calibration, time zero |
| gabapentin\_bulk-oralmixsf\_a\_syringe-25\_45;1722737;;calt45sf;stability | 1722737 | calt45sf | 15852 | 108.7 | 106.8 | 101.7 | calibration, time zero |
