# Supplementary material for: Stability of gabapentin in extemporaneously compounded oral suspensions
Source: PLoS One. 2017 Apr 17;12(4):e0175208. doi: 10.1371/journal.pone.0175208 (PMC5393583; doi:10.1371/journal.pone.0175208)
Supplement: S2 Appendix — Archive containing the HPLC stability results as browsable html pages. (ZIP) [file pone.0175208.s003.zip › gaba_s2_html_results/gabapentin/index.html?preparation=bulk-oralmixsf&lot=a&condition=syringe-25&time=60.html]

Stability Study Cruncher


### Preparation: bulk-oralmixsf, Lot: a, Condition: syringe-25, Time: 60

Assay (mg/mL): 105.4 ± 0.4 (n = 6);
Assay (%TZ): 98.6 ± 0.4 (n = 6).

| Input String | Area | Cal Id | Cal Slope | Assay | Assay TZ | Assay %TZ |  |
| --- | --- | --- | --- | --- | --- | --- | --- |
| gabapentin\_bulk-oralmixsf\_a\_syringe-25\_60;1662449;;calt45sf;stability | 1662449 | calt45sf | 15852 | 104.9 | 106.8 | 98.2 | calibration, time zero |
| gabapentin\_bulk-oralmixsf\_a\_syringe-25\_60;1662825;;calt45sf;stability | 1662825 | calt45sf | 15852 | 104.9 | 106.8 | 98.2 | calibration, time zero |
| gabapentin\_bulk-oralmixsf\_a\_syringe-25\_60;1675118;;calt45sf;stability | 1675118 | calt45sf | 15852 | 105.7 | 106.8 | 98.9 | calibration, time zero |
| gabapentin\_bulk-oralmixsf\_a\_syringe-25\_60;1675898;;calt45sf;stability | 1675898 | calt45sf | 15852 | 105.7 | 106.8 | 98.9 | calibration, time zero |
| gabapentin\_bulk-oralmixsf\_a\_syringe-25\_60;1673302;;calt45sf;stability | 1673302 | calt45sf | 15852 | 105.6 | 106.8 | 98.8 | calibration, time zero |
| gabapentin\_bulk-oralmixsf\_a\_syringe-25\_60;1673708;;calt45sf;stability | 1673708 | calt45sf | 15852 | 105.6 | 106.8 | 98.8 | calibration, time zero |
