# Supplementary material for: Stability of gabapentin in extemporaneously compounded oral suspensions
Source: PLoS One. 2017 Apr 17;12(4):e0175208. doi: 10.1371/journal.pone.0175208 (PMC5393583; doi:10.1371/journal.pone.0175208)
Supplement: S2 Appendix — Archive containing the HPLC stability results as browsable html pages. (ZIP) [file pone.0175208.s003.zip › gaba_s2_html_results/gabapentin/index.html?preparation=bulk-oralmixsf&lot=a&condition=syringe-25&time=75.html]

Stability Study Cruncher


### Preparation: bulk-oralmixsf, Lot: a, Condition: syringe-25, Time: 75

Assay (mg/mL): 109.9 ± 0.6 (n = 6);
Assay (%TZ): 102.9 ± 0.5 (n = 6).

| Input String | Area | Cal Id | Cal Slope | Assay | Assay TZ | Assay %TZ |  |
| --- | --- | --- | --- | --- | --- | --- | --- |
| gabapentin\_bulk-oralmixsf\_a\_syringe-25\_75;1730496;;calt45sf;stability | 1730496 | calt45sf | 15852 | 109.2 | 106.8 | 102.2 | calibration, time zero |
| gabapentin\_bulk-oralmixsf\_a\_syringe-25\_75;1739740;;calt45sf;stability | 1739740 | calt45sf | 15852 | 109.7 | 106.8 | 102.7 | calibration, time zero |
| gabapentin\_bulk-oralmixsf\_a\_syringe-25\_75;1737496;;calt45sf;stability | 1737496 | calt45sf | 15852 | 109.6 | 106.8 | 102.6 | calibration, time zero |
| gabapentin\_bulk-oralmixsf\_a\_syringe-25\_75;1740691;;calt45sf;stability | 1740691 | calt45sf | 15852 | 109.8 | 106.8 | 102.8 | calibration, time zero |
| gabapentin\_bulk-oralmixsf\_a\_syringe-25\_75;1756062;;calt45sf;stability | 1756062 | calt45sf | 15852 | 110.8 | 106.8 | 103.7 | calibration, time zero |
| gabapentin\_bulk-oralmixsf\_a\_syringe-25\_75;1748961;;calt45sf;stability | 1748961 | calt45sf | 15852 | 110.3 | 106.8 | 103.3 | calibration, time zero |
