# Supplementary material for: Stability of gabapentin in extemporaneously compounded oral suspensions
Source: PLoS One. 2017 Apr 17;12(4):e0175208. doi: 10.1371/journal.pone.0175208 (PMC5393583; doi:10.1371/journal.pone.0175208)
Supplement: S2 Appendix — Archive containing the HPLC stability results as browsable html pages. (ZIP) [file pone.0175208.s003.zip › gaba_s2_html_results/gabapentin/index.html?calibrationId=calt0sf.html]

Stability Study Cruncher


### Calibration Id: calt0sf

Slope: 15817 1/mg/mL (r2 = 0.99999, n = 18).

|  |  |  |  |  |  |  |  |  |  |  |  |  |  |  |  |  |  |  |  |  |  |  |  |  |  |  |  |  |  |  |  |  |  |  |  |  |  |  |  |  |  |  |  |  |  |  |  |  |  |  |  |  |  |  |  |  |
| --- | --- | --- | --- | --- | --- | --- | --- | --- | --- | --- | --- | --- | --- | --- | --- | --- | --- | --- | --- | --- | --- | --- | --- | --- | --- | --- | --- | --- | --- | --- | --- | --- | --- | --- | --- | --- | --- | --- | --- | --- | --- | --- | --- | --- | --- | --- | --- | --- | --- | --- | --- | --- | --- | --- | --- | --- |
| Input String | Conc | Area |||  |  |  |  |  |  |  |  |  |  |  |  |  |  |  |  |  |  |  |  |  |  |  |  |  |  |  |  |  |  |  |  |  |  |  |  |  |  |  |  |  |  |  |  |  |  |  |  |  |  |  |  |  |  |
| --- | --- | --- | --- | --- | --- | --- | --- | --- | --- | --- | --- | --- | --- | --- | --- | --- | --- | --- | --- | --- | --- | --- | --- | --- | --- | --- | --- | --- | --- | --- | --- | --- | --- | --- | --- | --- | --- | --- | --- | --- | --- | --- | --- | --- | --- | --- | --- | --- | --- | --- | --- | --- | --- |
| gabapentin\_STD00\_SF;0;0;calt0sf;calibration | 0.0 | 0 || gabapentin\_STD0.5\_SF;300236;18.584;calt0sf;calibration | 18.6 | 300236 || gabapentin\_STD1.0\_SF;591594;37.168;calt0sf;calibration | 37.2 | 591594 || gabapentin\_STD2.5\_SF;1470516;92.92;calt0sf;calibration | 92.9 | 1470516 || gabapentin\_STD3.75\_SF;2212656;139.38;calt0sf;calibration | 139.4 | 2212656 || gabapentin\_STD5.0\_SF;2936163;185.84;calt0sf;calibration | 185.8 | 2936163 || gabapentin\_STD00\_SF;0;0;calt0sf;calibration | 0.0 | 0 || gabapentin\_STD0.5\_SF;296967;18.584;calt0sf;calibration | 18.6 | 296967 || gabapentin\_STD1.0\_SF;592696;37.168;calt0sf;calibration | 37.2 | 592696 || gabapentin\_STD2.5\_SF;1468371;92.92;calt0sf;calibration | 92.9 | 1468371 || gabapentin\_STD3.75\_SF;2214359;139.38;calt0sf;calibration | 139.4 | 2214359 || gabapentin\_STD5.0\_SF;2929971;185.84;calt0sf;calibration | 185.8 | 2929971 || gabapentin\_STD00\_SF;0;0;calt0sf;calibration | 0.0 | 0 || gabapentin\_STD0.5\_SF;295482;18.584;calt0sf;calibration | 18.6 | 295482 || gabapentin\_STD1.0\_SF;592019;37.168;calt0sf;calibration | 37.2 | 592019 || gabapentin\_STD2.5\_SF;1463507;92.92;calt0sf;calibration | 92.9 | 1463507 || gabapentin\_STD3.75\_SF;2208638;139.38;calt0sf;calibration | 139.4 | 2208638 || gabapentin\_STD5.0\_SF;2935666;185.84;calt0sf;calibration | 185.8 | 2935666 |
