# Supplementary material for: Stability of gabapentin in extemporaneously compounded oral suspensions
Source: PLoS One. 2017 Apr 17;12(4):e0175208. doi: 10.1371/journal.pone.0175208 (PMC5393583; doi:10.1371/journal.pone.0175208)
Supplement: S2 Appendix — Archive containing the HPLC stability results as browsable html pages. (ZIP) [file pone.0175208.s003.zip › gaba_s2_html_results/gabapentin/index.html?preparation=bulk-oralmixsf&lot=a&condition=syringe-25&time=90.html]

Stability Study Cruncher


### Preparation: bulk-oralmixsf, Lot: a, Condition: syringe-25, Time: 90

Assay (mg/mL): 110.2 ± 1.1 (n = 6);
Assay (%TZ): 103.1 ± 1.1 (n = 6).

| Input String | Area | Cal Id | Cal Slope | Assay | Assay TZ | Assay %TZ |  |
| --- | --- | --- | --- | --- | --- | --- | --- |
| gabapentin\_bulk-oralmixsf\_a\_syringe-25\_90;1769567;;calt45sf;stability | 1769567 | calt45sf | 15852 | 111.6 | 106.8 | 104.5 | calibration, time zero |
| gabapentin\_bulk-oralmixsf\_a\_syringe-25\_90;1769618;;calt45sf;stability | 1769618 | calt45sf | 15852 | 111.6 | 106.8 | 104.5 | calibration, time zero |
| gabapentin\_bulk-oralmixsf\_a\_syringe-25\_90;1738900;;calt45sf;stability | 1738900 | calt45sf | 15852 | 109.7 | 106.8 | 102.7 | calibration, time zero |
| gabapentin\_bulk-oralmixsf\_a\_syringe-25\_90;1735014;;calt45sf;stability | 1735014 | calt45sf | 15852 | 109.4 | 106.8 | 102.4 | calibration, time zero |
| gabapentin\_bulk-oralmixsf\_a\_syringe-25\_90;1730059;;calt45sf;stability | 1730059 | calt45sf | 15852 | 109.1 | 106.8 | 102.1 | calibration, time zero |
| gabapentin\_bulk-oralmixsf\_a\_syringe-25\_90;1735135;;calt45sf;stability | 1735135 | calt45sf | 15852 | 109.5 | 106.8 | 102.4 | calibration, time zero |
