# Supplementary material for: Stability of gabapentin in extemporaneously compounded oral suspensions
Source: PLoS One. 2017 Apr 17;12(4):e0175208. doi: 10.1371/journal.pone.0175208 (PMC5393583; doi:10.1371/journal.pone.0175208)
Supplement: S2 Appendix — Archive containing the HPLC stability results as browsable html pages. (ZIP) [file pone.0175208.s003.zip › gaba_s2_html_results/gabapentin/index.html?preparation=tablet-oralmixsf&lot=a&condition=bottle-25&time=7.html]

Stability Study Cruncher


### Preparation: tablet-oralmixsf, Lot: a, Condition: bottle-25, Time: 7

Assay (mg/mL): 105.9 ± 2.1 (n = 6);
Assay (%TZ): 100.2 ± 2.0 (n = 6).

| Input String | Area | Cal Id | Cal Slope | Assay | Assay TZ | Assay %TZ |  |
| --- | --- | --- | --- | --- | --- | --- | --- |
| gabapentin\_tablet-oralmixsf\_a\_bottle-25\_7;1709417;;calt0sf;stability | 1709417 | calt0sf | 15817 | 108.1 | 105.7 | 102.3 | calibration, time zero |
| gabapentin\_tablet-oralmixsf\_a\_bottle-25\_7;1711269;;calt0sf;stability | 1711269 | calt0sf | 15817 | 108.2 | 105.7 | 102.4 | calibration, time zero |
| gabapentin\_tablet-oralmixsf\_a\_bottle-25\_7;1677030;;calt0sf;stability | 1677030 | calt0sf | 15817 | 106.0 | 105.7 | 100.3 | calibration, time zero |
| gabapentin\_tablet-oralmixsf\_a\_bottle-25\_7;1679969;;calt0sf;stability | 1679969 | calt0sf | 15817 | 106.2 | 105.7 | 100.5 | calibration, time zero |
| gabapentin\_tablet-oralmixsf\_a\_bottle-25\_7;1636118;;calt0sf;stability | 1636118 | calt0sf | 15817 | 103.4 | 105.7 | 97.9 | calibration, time zero |
| gabapentin\_tablet-oralmixsf\_a\_bottle-25\_7;1634774;;calt0sf;stability | 1634774 | calt0sf | 15817 | 103.4 | 105.7 | 97.8 | calibration, time zero |
