# Supplementary material for: Stability of gabapentin in extemporaneously compounded oral suspensions
Source: PLoS One. 2017 Apr 17;12(4):e0175208. doi: 10.1371/journal.pone.0175208 (PMC5393583; doi:10.1371/journal.pone.0175208)
Supplement: S2 Appendix — Archive containing the HPLC stability results as browsable html pages. (ZIP) [file pone.0175208.s003.zip › gaba_s2_html_results/gabapentin/index.html?preparation=tablet-oralmixsf&lot=a&condition=syringe-25&time=75.html]

Stability Study Cruncher


### Preparation: tablet-oralmixsf, Lot: a, Condition: syringe-25, Time: 75

Assay (mg/mL): 110.0 ± 1.0 (n = 6);
Assay (%TZ): 104.1 ± 1.0 (n = 6).

| Input String | Area | Cal Id | Cal Slope | Assay | Assay TZ | Assay %TZ |  |
| --- | --- | --- | --- | --- | --- | --- | --- |
| gabapentin\_tablet-oralmixsf\_a\_syringe-25\_75;1735617;;calt45sf;stability | 1735617 | calt45sf | 15852 | 109.5 | 105.7 | 103.6 | calibration, time zero |
| gabapentin\_tablet-oralmixsf\_a\_syringe-25\_75;1739751;;calt45sf;stability | 1739751 | calt45sf | 15852 | 109.7 | 105.7 | 103.8 | calibration, time zero |
| gabapentin\_tablet-oralmixsf\_a\_syringe-25\_75;1728396;;calt45sf;stability | 1728396 | calt45sf | 15852 | 109.0 | 105.7 | 103.2 | calibration, time zero |
| gabapentin\_tablet-oralmixsf\_a\_syringe-25\_75;1731838;;calt45sf;stability | 1731838 | calt45sf | 15852 | 109.2 | 105.7 | 103.4 | calibration, time zero |
| gabapentin\_tablet-oralmixsf\_a\_syringe-25\_75;1753891;;calt45sf;stability | 1753891 | calt45sf | 15852 | 110.6 | 105.7 | 104.7 | calibration, time zero |
| gabapentin\_tablet-oralmixsf\_a\_syringe-25\_75;1771655;;calt45sf;stability | 1771655 | calt45sf | 15852 | 111.8 | 105.7 | 105.7 | calibration, time zero |
