# Supplementary material for: Stability of gabapentin in extemporaneously compounded oral suspensions
Source: PLoS One. 2017 Apr 17;12(4):e0175208. doi: 10.1371/journal.pone.0175208 (PMC5393583; doi:10.1371/journal.pone.0175208)
Supplement: S2 Appendix — Archive containing the HPLC stability results as browsable html pages. (ZIP) [file pone.0175208.s003.zip › gaba_s2_html_results/gabapentin/index.html?preparation=tablet-oralmixsf&lot=a&condition=bottle-25&time=30.html]

Stability Study Cruncher


### Preparation: tablet-oralmixsf, Lot: a, Condition: bottle-25, Time: 30

Assay (mg/mL): 112.2 ± 4.4 (n = 6);
Assay (%TZ): 106.2 ± 4.1 (n = 6).

| Input String | Area | Cal Id | Cal Slope | Assay | Assay TZ | Assay %TZ |  |
| --- | --- | --- | --- | --- | --- | --- | --- |
| gabapentin\_tablet-oralmixsf\_a\_bottle-25\_30;1834535;;calt0sf;stability | 1834535 | calt0sf | 15817 | 116.0 | 105.7 | 109.7 | calibration, time zero |
| gabapentin\_tablet-oralmixsf\_a\_bottle-25\_30;1839223;;calt0sf;stability | 1839223 | calt0sf | 15817 | 116.3 | 105.7 | 110.0 | calibration, time zero |
| gabapentin\_tablet-oralmixsf\_a\_bottle-25\_30;1688485;;calt0sf;stability | 1688485 | calt0sf | 15817 | 106.8 | 105.7 | 101.0 | calibration, time zero |
| gabapentin\_tablet-oralmixsf\_a\_bottle-25\_30;1688641;;calt0sf;stability | 1688641 | calt0sf | 15817 | 106.8 | 105.7 | 101.0 | calibration, time zero |
| gabapentin\_tablet-oralmixsf\_a\_bottle-25\_30;1797374;;calt0sf;stability | 1797374 | calt0sf | 15817 | 113.6 | 105.7 | 107.5 | calibration, time zero |
| gabapentin\_tablet-oralmixsf\_a\_bottle-25\_30;1801813;;calt0sf;stability | 1801813 | calt0sf | 15817 | 113.9 | 105.7 | 107.8 | calibration, time zero |
