# Supplementary material for: Stability of gabapentin in extemporaneously compounded oral suspensions
Source: PLoS One. 2017 Apr 17;12(4):e0175208. doi: 10.1371/journal.pone.0175208 (PMC5393583; doi:10.1371/journal.pone.0175208)
Supplement: S2 Appendix — Archive containing the HPLC stability results as browsable html pages. (ZIP) [file pone.0175208.s003.zip › gaba_s2_html_results/gabapentin/index.html?preparation=tablet-oralmixsf&lot=a&condition=bottle-25&time=45.html]

Stability Study Cruncher


### Preparation: tablet-oralmixsf, Lot: a, Condition: bottle-25, Time: 45

Assay (mg/mL): 107.1 ± 0.9 (n = 6);
Assay (%TZ): 101.4 ± 0.8 (n = 6).

| Input String | Area | Cal Id | Cal Slope | Assay | Assay TZ | Assay %TZ |  |
| --- | --- | --- | --- | --- | --- | --- | --- |
| gabapentin\_tablet-oralmixsf\_a\_bottle-25\_45;1712267;;calt45sf;stability | 1712267 | calt45sf | 15852 | 108.0 | 105.7 | 102.2 | calibration, time zero |
| gabapentin\_tablet-oralmixsf\_a\_bottle-25\_45;1719057;;calt45sf;stability | 1719057 | calt45sf | 15852 | 108.4 | 105.7 | 102.6 | calibration, time zero |
| gabapentin\_tablet-oralmixsf\_a\_bottle-25\_45;1690451;;calt45sf;stability | 1690451 | calt45sf | 15852 | 106.6 | 105.7 | 100.9 | calibration, time zero |
| gabapentin\_tablet-oralmixsf\_a\_bottle-25\_45;1688010;;calt45sf;stability | 1688010 | calt45sf | 15852 | 106.5 | 105.7 | 100.8 | calibration, time zero |
| gabapentin\_tablet-oralmixsf\_a\_bottle-25\_45;1690012;;calt45sf;stability | 1690012 | calt45sf | 15852 | 106.6 | 105.7 | 100.9 | calibration, time zero |
| gabapentin\_tablet-oralmixsf\_a\_bottle-25\_45;1690260;;calt45sf;stability | 1690260 | calt45sf | 15852 | 106.6 | 105.7 | 100.9 | calibration, time zero |
