# Supplementary material for: Stability of gabapentin in extemporaneously compounded oral suspensions
Source: PLoS One. 2017 Apr 17;12(4):e0175208. doi: 10.1371/journal.pone.0175208 (PMC5393583; doi:10.1371/journal.pone.0175208)
Supplement: S2 Appendix — Archive containing the HPLC stability results as browsable html pages. (ZIP) [file pone.0175208.s003.zip › gaba_s2_html_results/gabapentin/index.html?calibrationId=calt45sf.html]

Stability Study Cruncher


### Calibration Id: calt45sf

Slope: 15852 1/mg/mL (r2 = 1.00000, n = 18).

|  |  |  |  |  |  |  |  |  |  |  |  |  |  |  |  |  |  |  |  |  |  |  |  |  |  |  |  |  |  |  |  |  |  |  |  |  |  |  |  |  |  |  |  |  |  |  |  |  |  |  |  |  |  |  |  |  |
| --- | --- | --- | --- | --- | --- | --- | --- | --- | --- | --- | --- | --- | --- | --- | --- | --- | --- | --- | --- | --- | --- | --- | --- | --- | --- | --- | --- | --- | --- | --- | --- | --- | --- | --- | --- | --- | --- | --- | --- | --- | --- | --- | --- | --- | --- | --- | --- | --- | --- | --- | --- | --- | --- | --- | --- | --- |
| Input String | Conc | Area |||  |  |  |  |  |  |  |  |  |  |  |  |  |  |  |  |  |  |  |  |  |  |  |  |  |  |  |  |  |  |  |  |  |  |  |  |  |  |  |  |  |  |  |  |  |  |  |  |  |  |  |  |  |  |
| --- | --- | --- | --- | --- | --- | --- | --- | --- | --- | --- | --- | --- | --- | --- | --- | --- | --- | --- | --- | --- | --- | --- | --- | --- | --- | --- | --- | --- | --- | --- | --- | --- | --- | --- | --- | --- | --- | --- | --- | --- | --- | --- | --- | --- | --- | --- | --- | --- | --- | --- | --- | --- | --- |
| gabapentin\_STD00\_SF;0;0;calt45sf;calibration | 0.0 | 0 || gabapentin\_STD0.5\_SF;291232;18.584;calt45sf;calibration | 18.6 | 291232 || gabapentin\_STD1.0\_SF;594424;37.168;calt45sf;calibration | 37.2 | 594424 || gabapentin\_STD2.5\_SF;1472905;92.92;calt45sf;calibration | 92.9 | 1472905 || gabapentin\_STD3.75\_SF;2208409;139.38;calt45sf;calibration | 139.4 | 2208409 || gabapentin\_STD5.0\_SF;2946883;185.84;calt45sf;calibration | 185.8 | 2946883 || gabapentin\_STD00\_SF;0;0;calt45sf;calibration | 0.0 | 0 || gabapentin\_STD0.5\_SF;295048;18.584;calt45sf;calibration | 18.6 | 295048 || gabapentin\_STD1.0\_SF;593542;37.168;calt45sf;calibration | 37.2 | 593542 || gabapentin\_STD2.5\_SF;1471925;92.92;calt45sf;calibration | 92.9 | 1471925 || gabapentin\_STD3.75\_SF;2209676;139.38;calt45sf;calibration | 139.4 | 2209676 || gabapentin\_STD5.0\_SF;2948146;185.84;calt45sf;calibration | 185.8 | 2948146 || gabapentin\_STD00\_SF;0;0;calt45sf;calibration | 0.0 | 0 || gabapentin\_STD0.5\_SF;294858;18.584;calt45sf;calibration | 18.6 | 294858 || gabapentin\_STD1.0\_SF;593045;37.168;calt45sf;calibration | 37.2 | 593045 || gabapentin\_STD2.5\_SF;1463329;92.92;calt45sf;calibration | 92.9 | 1463329 || gabapentin\_STD3.75\_SF;2207304;139.38;calt45sf;calibration | 139.4 | 2207304 || gabapentin\_STD5.0\_SF;2948503;185.84;calt45sf;calibration | 185.8 | 2948503 |
