# Supplementary material for: Stability of gabapentin in extemporaneously compounded oral suspensions
Source: PLoS One. 2017 Apr 17;12(4):e0175208. doi: 10.1371/journal.pone.0175208 (PMC5393583; doi:10.1371/journal.pone.0175208)
Supplement: S2 Appendix — Archive containing the HPLC stability results as browsable html pages. (ZIP) [file pone.0175208.s003.zip › gaba_s2_html_results/gabapentin/index.html?preparation=tablet-oralmixsf&lot=a&condition=bottle-25&time=60.html]

Stability Study Cruncher


### Preparation: tablet-oralmixsf, Lot: a, Condition: bottle-25, Time: 60

Assay (mg/mL): 105.2 ± 0.3 (n = 6);
Assay (%TZ): 99.5 ± 0.3 (n = 6).

| Input String | Area | Cal Id | Cal Slope | Assay | Assay TZ | Assay %TZ |  |
| --- | --- | --- | --- | --- | --- | --- | --- |
| gabapentin\_tablet-oralmixsf\_a\_bottle-25\_60;1668674;;calt45sf;stability | 1668674 | calt45sf | 15852 | 105.3 | 105.7 | 99.6 | calibration, time zero |
| gabapentin\_tablet-oralmixsf\_a\_bottle-25\_60;1664548;;calt45sf;stability | 1664548 | calt45sf | 15852 | 105.0 | 105.7 | 99.4 | calibration, time zero |
| gabapentin\_tablet-oralmixsf\_a\_bottle-25\_60;1664874;;calt45sf;stability | 1664874 | calt45sf | 15852 | 105.0 | 105.7 | 99.4 | calibration, time zero |
| gabapentin\_tablet-oralmixsf\_a\_bottle-25\_60;1661208;;calt45sf;stability | 1661208 | calt45sf | 15852 | 104.8 | 105.7 | 99.2 | calibration, time zero |
| gabapentin\_tablet-oralmixsf\_a\_bottle-25\_60;1671298;;calt45sf;stability | 1671298 | calt45sf | 15852 | 105.4 | 105.7 | 99.8 | calibration, time zero |
| gabapentin\_tablet-oralmixsf\_a\_bottle-25\_60;1672737;;calt45sf;stability | 1672737 | calt45sf | 15852 | 105.5 | 105.7 | 99.8 | calibration, time zero |
