# Supplementary material for: Stability of gabapentin in extemporaneously compounded oral suspensions
Source: PLoS One. 2017 Apr 17;12(4):e0175208. doi: 10.1371/journal.pone.0175208 (PMC5393583; doi:10.1371/journal.pone.0175208)
Supplement: S2 Appendix — Archive containing the HPLC stability results as browsable html pages. (ZIP) [file pone.0175208.s003.zip › gaba_s2_html_results/gabapentin/index.html?preparation=tablet-oralmixsf&lot=a&condition=bottle-25&time=75.html]

Stability Study Cruncher


### Preparation: tablet-oralmixsf, Lot: a, Condition: bottle-25, Time: 75

Assay (mg/mL): 112.0 ± 0.3 (n = 6);
Assay (%TZ): 105.9 ± 0.3 (n = 6).

| Input String | Area | Cal Id | Cal Slope | Assay | Assay TZ | Assay %TZ |  |
| --- | --- | --- | --- | --- | --- | --- | --- |
| gabapentin\_tablet-oralmixsf\_a\_bottle-25\_75;1781446;;calt45sf;stability | 1781446 | calt45sf | 15852 | 112.4 | 105.7 | 106.3 | calibration, time zero |
| gabapentin\_tablet-oralmixsf\_a\_bottle-25\_75;1770718;;calt45sf;stability | 1770718 | calt45sf | 15852 | 111.7 | 105.7 | 105.7 | calibration, time zero |
| gabapentin\_tablet-oralmixsf\_a\_bottle-25\_75;1775490;;calt45sf;stability | 1775490 | calt45sf | 15852 | 112.0 | 105.7 | 106.0 | calibration, time zero |
| gabapentin\_tablet-oralmixsf\_a\_bottle-25\_75;1776831;;calt45sf;stability | 1776831 | calt45sf | 15852 | 112.1 | 105.7 | 106.1 | calibration, time zero |
| gabapentin\_tablet-oralmixsf\_a\_bottle-25\_75;1776925;;calt45sf;stability | 1776925 | calt45sf | 15852 | 112.1 | 105.7 | 106.1 | calibration, time zero |
| gabapentin\_tablet-oralmixsf\_a\_bottle-25\_75;1769058;;calt45sf;stability | 1769058 | calt45sf | 15852 | 111.6 | 105.7 | 105.6 | calibration, time zero |
