# Supplementary material for: Stability of gabapentin in extemporaneously compounded oral suspensions
Source: PLoS One. 2017 Apr 17;12(4):e0175208. doi: 10.1371/journal.pone.0175208 (PMC5393583; doi:10.1371/journal.pone.0175208)
Supplement: S2 Appendix — Archive containing the HPLC stability results as browsable html pages. (ZIP) [file pone.0175208.s003.zip › gaba_s2_html_results/gabapentin/index.html?preparation=tablet-oralmixsf&lot=a&condition=bottle-25&time=90.html]

Stability Study Cruncher


### Preparation: tablet-oralmixsf, Lot: a, Condition: bottle-25, Time: 90

Assay (mg/mL): 110.7 ± 0.2 (n = 6);
Assay (%TZ): 104.7 ± 0.2 (n = 6).

| Input String | Area | Cal Id | Cal Slope | Assay | Assay TZ | Assay %TZ |  |
| --- | --- | --- | --- | --- | --- | --- | --- |
| gabapentin\_tablet-oralmixsf\_a\_bottle-25\_90;1753909;;calt45sf;stability | 1753909 | calt45sf | 15852 | 110.6 | 105.7 | 104.7 | calibration, time zero |
| gabapentin\_tablet-oralmixsf\_a\_bottle-25\_90;1755854;;calt45sf;stability | 1755854 | calt45sf | 15852 | 110.8 | 105.7 | 104.8 | calibration, time zero |
| gabapentin\_tablet-oralmixsf\_a\_bottle-25\_90;1749339;;calt45sf;stability | 1749339 | calt45sf | 15852 | 110.4 | 105.7 | 104.4 | calibration, time zero |
| gabapentin\_tablet-oralmixsf\_a\_bottle-25\_90;1756431;;calt45sf;stability | 1756431 | calt45sf | 15852 | 110.8 | 105.7 | 104.8 | calibration, time zero |
| gabapentin\_tablet-oralmixsf\_a\_bottle-25\_90;1758584;;calt45sf;stability | 1758584 | calt45sf | 15852 | 110.9 | 105.7 | 105.0 | calibration, time zero |
| gabapentin\_tablet-oralmixsf\_a\_bottle-25\_90;1754093;;calt45sf;stability | 1754093 | calt45sf | 15852 | 110.7 | 105.7 | 104.7 | calibration, time zero |
