# Supplementary material for: Stability of gabapentin in extemporaneously compounded oral suspensions
Source: PLoS One. 2017 Apr 17;12(4):e0175208. doi: 10.1371/journal.pone.0175208 (PMC5393583; doi:10.1371/journal.pone.0175208)
Supplement: S2 Appendix — Archive containing the HPLC stability results as browsable html pages. (ZIP) [file pone.0175208.s003.zip › gaba_s2_html_results/gabapentin/index.html?preparation=tablet-oralmixsf&lot=a&condition=syringe-25&time=7.html]

Stability Study Cruncher


### Preparation: tablet-oralmixsf, Lot: a, Condition: syringe-25, Time: 7

Assay (mg/mL): 107.8 ± 0.7 (n = 6);
Assay (%TZ): 102.0 ± 0.6 (n = 6).

| Input String | Area | Cal Id | Cal Slope | Assay | Assay TZ | Assay %TZ |  |
| --- | --- | --- | --- | --- | --- | --- | --- |
| gabapentin\_tablet-oralmixsf\_a\_syringe-25\_7;1694219;;calt0sf;stability | 1694219 | calt0sf | 15817 | 107.1 | 105.7 | 101.3 | calibration, time zero |
| gabapentin\_tablet-oralmixsf\_a\_syringe-25\_7;1693720;;calt0sf;stability | 1693720 | calt0sf | 15817 | 107.1 | 105.7 | 101.3 | calibration, time zero |
| gabapentin\_tablet-oralmixsf\_a\_syringe-25\_7;1713512;;calt0sf;stability | 1713512 | calt0sf | 15817 | 108.3 | 105.7 | 102.5 | calibration, time zero |
| gabapentin\_tablet-oralmixsf\_a\_syringe-25\_7;1719958;;calt0sf;stability | 1719958 | calt0sf | 15817 | 108.7 | 105.7 | 102.9 | calibration, time zero |
| gabapentin\_tablet-oralmixsf\_a\_syringe-25\_7;1702977;;calt0sf;stability | 1702977 | calt0sf | 15817 | 107.7 | 105.7 | 101.9 | calibration, time zero |
| gabapentin\_tablet-oralmixsf\_a\_syringe-25\_7;1704343;;calt0sf;stability | 1704343 | calt0sf | 15817 | 107.8 | 105.7 | 102.0 | calibration, time zero |
