# Supplementary material for: Stability of gabapentin in extemporaneously compounded oral suspensions
Source: PLoS One. 2017 Apr 17;12(4):e0175208. doi: 10.1371/journal.pone.0175208 (PMC5393583; doi:10.1371/journal.pone.0175208)
Supplement: S2 Appendix — Archive containing the HPLC stability results as browsable html pages. (ZIP) [file pone.0175208.s003.zip › gaba_s2_html_results/gabapentin/index.html?preparation=tablet-oralmixsf&lot=a&condition=syringe-25&time=14.html]

Stability Study Cruncher


### Preparation: tablet-oralmixsf, Lot: a, Condition: syringe-25, Time: 14

Assay (mg/mL): 112.8 ± 1.6 (n = 6);
Assay (%TZ): 106.8 ± 1.5 (n = 6).

| Input String | Area | Cal Id | Cal Slope | Assay | Assay TZ | Assay %TZ |  |
| --- | --- | --- | --- | --- | --- | --- | --- |
| gabapentin\_tablet-oralmixsf\_a\_syringe-25\_14;1813289;;calt0sf;stability | 1813289 | calt0sf | 15817 | 114.6 | 105.7 | 108.5 | calibration, time zero |
| gabapentin\_tablet-oralmixsf\_a\_syringe-25\_14;1814032;;calt0sf;stability | 1814032 | calt0sf | 15817 | 114.7 | 105.7 | 108.5 | calibration, time zero |
| gabapentin\_tablet-oralmixsf\_a\_syringe-25\_14;1756388;;calt0sf;stability | 1756388 | calt0sf | 15817 | 111.0 | 105.7 | 105.1 | calibration, time zero |
| gabapentin\_tablet-oralmixsf\_a\_syringe-25\_14;1757982;;calt0sf;stability | 1757982 | calt0sf | 15817 | 111.1 | 105.7 | 105.2 | calibration, time zero |
| gabapentin\_tablet-oralmixsf\_a\_syringe-25\_14;1783954;;calt0sf;stability | 1783954 | calt0sf | 15817 | 112.8 | 105.7 | 106.7 | calibration, time zero |
| gabapentin\_tablet-oralmixsf\_a\_syringe-25\_14;1784060;;calt0sf;stability | 1784060 | calt0sf | 15817 | 112.8 | 105.7 | 106.7 | calibration, time zero |
