# Supplementary material for: Stability of gabapentin in extemporaneously compounded oral suspensions
Source: PLoS One. 2017 Apr 17;12(4):e0175208. doi: 10.1371/journal.pone.0175208 (PMC5393583; doi:10.1371/journal.pone.0175208)
Supplement: S2 Appendix — Archive containing the HPLC stability results as browsable html pages. (ZIP) [file pone.0175208.s003.zip › gaba_s2_html_results/gabapentin/index.html?preparation=tablet-oralmix&lot=a.html]

Stability Study Cruncher


### Preparation: tablet-oralmix, Lot: a

Assay: 101.3 ± 1.9 mg/mL (n = 12).

| Input String | Area | Cal Id | Cal Slope | Assay |  |
| --- | --- | --- | --- | --- | --- |
| gabapentin\_tablet-oralmix\_a\_bottle-25;1759840;;calt0om;time zero | 1759840 | calt0om | 16864 | 104.4 | calibration |
| gabapentin\_tablet-oralmix\_a\_bottle-25;1757548;;calt0om;time zero | 1757548 | calt0om | 16864 | 104.2 | calibration |
| gabapentin\_tablet-oralmix\_a\_bottle-25;1680712;;calt0om;time zero | 1680712 | calt0om | 16864 | 99.7 | calibration |
| gabapentin\_tablet-oralmix\_a\_bottle-25;1680607;;calt0om;time zero | 1680607 | calt0om | 16864 | 99.7 | calibration |
| gabapentin\_tablet-oralmix\_a\_bottle-25;1725628;;calt0om;time zero | 1725628 | calt0om | 16864 | 102.3 | calibration |
| gabapentin\_tablet-oralmix\_a\_bottle-25;1719414;;calt0om;time zero | 1719414 | calt0om | 16864 | 102.0 | calibration |
| gabapentin\_tablet-oralmix\_a\_syringe-25;1672274;;calt0om;time zero | 1672274 | calt0om | 16864 | 99.2 | calibration |
| gabapentin\_tablet-oralmix\_a\_syringe-25;1666896;;calt0om;time zero | 1666896 | calt0om | 16864 | 98.8 | calibration |
| gabapentin\_tablet-oralmix\_a\_syringe-25;1725603;;calt0om;time zero | 1725603 | calt0om | 16864 | 102.3 | calibration |
| gabapentin\_tablet-oralmix\_a\_syringe-25;1726248;;calt0om;time zero | 1726248 | calt0om | 16864 | 102.4 | calibration |
| gabapentin\_tablet-oralmix\_a\_syringe-25;1686371;;calt0om;time zero | 1686371 | calt0om | 16864 | 100.0 | calibration |
| gabapentin\_tablet-oralmix\_a\_syringe-25;1691250;;calt0om;time zero | 1691250 | calt0om | 16864 | 100.3 | calibration |
