# Supplementary material for: Stability of gabapentin in extemporaneously compounded oral suspensions
Source: PLoS One. 2017 Apr 17;12(4):e0175208. doi: 10.1371/journal.pone.0175208 (PMC5393583; doi:10.1371/journal.pone.0175208)
Supplement: S2 Appendix — Archive containing the HPLC stability results as browsable html pages. (ZIP) [file pone.0175208.s003.zip › gaba_s2_html_results/gabapentin/index.html?preparation=tablet-oralmixsf&lot=a&condition=syringe-25&time=30.html]

Stability Study Cruncher


### Preparation: tablet-oralmixsf, Lot: a, Condition: syringe-25, Time: 30

Assay (mg/mL): 110.1 ± 1.9 (n = 6);
Assay (%TZ): 104.2 ± 1.8 (n = 6).

| Input String | Area | Cal Id | Cal Slope | Assay | Assay TZ | Assay %TZ |  |
| --- | --- | --- | --- | --- | --- | --- | --- |
| gabapentin\_tablet-oralmixsf\_a\_syringe-25\_30;1703192;;calt0sf;stability | 1703192 | calt0sf | 15817 | 107.7 | 105.7 | 101.9 | calibration, time zero |
| gabapentin\_tablet-oralmixsf\_a\_syringe-25\_30;1703927;;calt0sf;stability | 1703927 | calt0sf | 15817 | 107.7 | 105.7 | 101.9 | calibration, time zero |
| gabapentin\_tablet-oralmixsf\_a\_syringe-25\_30;1754476;;calt0sf;stability | 1754476 | calt0sf | 15817 | 110.9 | 105.7 | 105.0 | calibration, time zero |
| gabapentin\_tablet-oralmixsf\_a\_syringe-25\_30;1754193;;calt0sf;stability | 1754193 | calt0sf | 15817 | 110.9 | 105.7 | 104.9 | calibration, time zero |
| gabapentin\_tablet-oralmixsf\_a\_syringe-25\_30;1767036;;calt0sf;stability | 1767036 | calt0sf | 15817 | 111.7 | 105.7 | 105.7 | calibration, time zero |
| gabapentin\_tablet-oralmixsf\_a\_syringe-25\_30;1766110;;calt0sf;stability | 1766110 | calt0sf | 15817 | 111.7 | 105.7 | 105.6 | calibration, time zero |
