# Supplementary material for: Stability of gabapentin in extemporaneously compounded oral suspensions
Source: PLoS One. 2017 Apr 17;12(4):e0175208. doi: 10.1371/journal.pone.0175208 (PMC5393583; doi:10.1371/journal.pone.0175208)
Supplement: S2 Appendix — Archive containing the HPLC stability results as browsable html pages. (ZIP) [file pone.0175208.s003.zip › gaba_s2_html_results/gabapentin/index.html?preparation=tablet-oralmixsf&lot=a&condition=syringe-25&time=45.html]

Stability Study Cruncher


### Preparation: tablet-oralmixsf, Lot: a, Condition: syringe-25, Time: 45

Assay (mg/mL): 105.7 ± 0.6 (n = 6);
Assay (%TZ): 100.0 ± 0.5 (n = 6).

| Input String | Area | Cal Id | Cal Slope | Assay | Assay TZ | Assay %TZ |  |
| --- | --- | --- | --- | --- | --- | --- | --- |
| gabapentin\_tablet-oralmixsf\_a\_syringe-25\_45;1665115;;calt45sf;stability | 1665115 | calt45sf | 15852 | 105.0 | 105.7 | 99.4 | calibration, time zero |
| gabapentin\_tablet-oralmixsf\_a\_syringe-25\_45;1665179;;calt45sf;stability | 1665179 | calt45sf | 15852 | 105.0 | 105.7 | 99.4 | calibration, time zero |
| gabapentin\_tablet-oralmixsf\_a\_syringe-25\_45;1674827;;calt45sf;stability | 1674827 | calt45sf | 15852 | 105.7 | 105.7 | 100.0 | calibration, time zero |
| gabapentin\_tablet-oralmixsf\_a\_syringe-25\_45;1676395;;calt45sf;stability | 1676395 | calt45sf | 15852 | 105.7 | 105.7 | 100.1 | calibration, time zero |
| gabapentin\_tablet-oralmixsf\_a\_syringe-25\_45;1684647;;calt45sf;stability | 1684647 | calt45sf | 15852 | 106.3 | 105.7 | 100.6 | calibration, time zero |
| gabapentin\_tablet-oralmixsf\_a\_syringe-25\_45;1685073;;calt45sf;stability | 1685073 | calt45sf | 15852 | 106.3 | 105.7 | 100.6 | calibration, time zero |
