# Supplementary material for: Stability of gabapentin in extemporaneously compounded oral suspensions
Source: PLoS One. 2017 Apr 17;12(4):e0175208. doi: 10.1371/journal.pone.0175208 (PMC5393583; doi:10.1371/journal.pone.0175208)
Supplement: S2 Appendix — Archive containing the HPLC stability results as browsable html pages. (ZIP) [file pone.0175208.s003.zip › gaba_s2_html_results/gabapentin/index.html?preparation=tablet-oralmixsf&lot=a&condition=syringe-25&time=60.html]

Stability Study Cruncher


### Preparation: tablet-oralmixsf, Lot: a, Condition: syringe-25, Time: 60

Assay (mg/mL): 106.0 ± 0.3 (n = 6);
Assay (%TZ): 100.3 ± 0.3 (n = 6).

| Input String | Area | Cal Id | Cal Slope | Assay | Assay TZ | Assay %TZ |  |
| --- | --- | --- | --- | --- | --- | --- | --- |
| gabapentin\_tablet-oralmixsf\_a\_syringe-25\_60;1686438;;calt45sf;stability | 1686438 | calt45sf | 15852 | 106.4 | 105.7 | 100.7 | calibration, time zero |
| gabapentin\_tablet-oralmixsf\_a\_syringe-25\_60;1688711;;calt45sf;stability | 1688711 | calt45sf | 15852 | 106.5 | 105.7 | 100.8 | calibration, time zero |
| gabapentin\_tablet-oralmixsf\_a\_syringe-25\_60;1678720;;calt45sf;stability | 1678720 | calt45sf | 15852 | 105.9 | 105.7 | 100.2 | calibration, time zero |
| gabapentin\_tablet-oralmixsf\_a\_syringe-25\_60;1679970;;calt45sf;stability | 1679970 | calt45sf | 15852 | 106.0 | 105.7 | 100.3 | calibration, time zero |
| gabapentin\_tablet-oralmixsf\_a\_syringe-25\_60;1674688;;calt45sf;stability | 1674688 | calt45sf | 15852 | 105.6 | 105.7 | 100.0 | calibration, time zero |
| gabapentin\_tablet-oralmixsf\_a\_syringe-25\_60;1676760;;calt45sf;stability | 1676760 | calt45sf | 15852 | 105.8 | 105.7 | 100.1 | calibration, time zero |
