# Supplementary material for: Stability of gabapentin in extemporaneously compounded oral suspensions
Source: PLoS One. 2017 Apr 17;12(4):e0175208. doi: 10.1371/journal.pone.0175208 (PMC5393583; doi:10.1371/journal.pone.0175208)
Supplement: S2 Appendix — Archive containing the HPLC stability results as browsable html pages. (ZIP) [file pone.0175208.s003.zip › gaba_s2_html_results/gabapentin/index.html?preparation=tablet-oralmixsf&lot=a&condition=syringe-25&time=90.html]

Stability Study Cruncher


### Preparation: tablet-oralmixsf, Lot: a, Condition: syringe-25, Time: 90

Assay (mg/mL): 110.0 ± 1.6 (n = 6);
Assay (%TZ): 104.1 ± 1.5 (n = 6).

| Input String | Area | Cal Id | Cal Slope | Assay | Assay TZ | Assay %TZ |  |
| --- | --- | --- | --- | --- | --- | --- | --- |
| gabapentin\_tablet-oralmixsf\_a\_syringe-25\_90;1714459;;calt45sf;stability | 1714459 | calt45sf | 15852 | 108.2 | 105.7 | 102.3 | calibration, time zero |
| gabapentin\_tablet-oralmixsf\_a\_syringe-25\_90;1709686;;calt45sf;stability | 1709686 | calt45sf | 15852 | 107.8 | 105.7 | 102.0 | calibration, time zero |
| gabapentin\_tablet-oralmixsf\_a\_syringe-25\_90;1759977;;calt45sf;stability | 1759977 | calt45sf | 15852 | 111.0 | 105.7 | 105.0 | calibration, time zero |
| gabapentin\_tablet-oralmixsf\_a\_syringe-25\_90;1766287;;calt45sf;stability | 1766287 | calt45sf | 15852 | 111.4 | 105.7 | 105.4 | calibration, time zero |
| gabapentin\_tablet-oralmixsf\_a\_syringe-25\_90;1757511;;calt45sf;stability | 1757511 | calt45sf | 15852 | 110.9 | 105.7 | 104.9 | calibration, time zero |
| gabapentin\_tablet-oralmixsf\_a\_syringe-25\_90;1758831;;calt45sf;stability | 1758831 | calt45sf | 15852 | 110.9 | 105.7 | 105.0 | calibration, time zero |
